# The effect of spatial randomness on the average fixation time of mutants

**DOI:** 10.1371/journal.pcbi.1005864

**Published:** 2017-11-27

**Authors:** Suzan Farhang-Sardroodi, Amir H. Darooneh, Moladad Nikbakht, Natalia L. Komarova, Mohammad Kohandel

**Affiliations:** 1 Department of Physics, University of Zanjan, Zanjan, Iran; 2 Department of Mathematics, University of California Irvine, Irvine, California, United States of America; 3 Department of Applied Mathematics, University of Waterloo, Waterloo, Ontario, Canada; University of New South Wales, AUSTRALIA

## Abstract

The mean conditional fixation time of a mutant is an important measure of stochastic population dynamics, widely studied in ecology and evolution. Here, we investigate the effect of spatial randomness on the mean conditional fixation time of mutants in a constant population of cells, *N*. Specifically, we assume that fitness values of wild type cells and mutants at different locations come from given probability distributions and do not change in time. We study spatial arrangements of cells on regular graphs with different degrees, from the circle to the complete graph, and vary assumptions on the fitness probability distributions. Some examples include: identical probability distributions for wild types and mutants; cases when only one of the cell types has random fitness values while the other has deterministic fitness; and cases where the mutants are advantaged or disadvantaged. Using analytical calculations and stochastic numerical simulations, we find that randomness has a strong impact on fixation time. In the case of complete graphs, randomness accelerates mutant fixation for all population sizes, and in the case of circular graphs, randomness delays mutant fixation for *N* larger than a threshold value (for small values of *N*, different behaviors are observed depending on the fitness distribution functions). These results emphasize fundamental differences in population dynamics under different assumptions on cell connectedness. They are explained by the existence of randomly occurring “dead zones” that can significantly delay fixation on networks with low connectivity; and by the existence of randomly occurring “lucky zones” that can facilitate fixation on networks of high connectivity. Results for death-birth and birth-death formulations of the Moran process, as well as for the (haploid) Wright Fisher model are presented.

## Author summary

We study the influence of randomness on evolutionary dynamics, assuming that a newly arising mutant may experience a different set of environments compared to the wild type. We calculate the mean conditional fixation time of the mutant under different assumptions on spatial interactions, and show that randomness has a strong impact on the fixation time. In particular, it delays the fixation of mutants on 1D circles and accelerates it on complete graphs (the so called mass action, or complete mixing, model). This result holds for advantageous, disadvantageous, and neutral (on average) mutants. The reason for this pattern is quite intuitive: in a rigid, 1D structure, randomness can by chance put a “roadblock” and disrupt mutant spread, causing significant delay. In higher dimensions, there are many ways for a mutant to spread, and it is difficult to block all of them by chance; on the other hand, randomness can enhance fixation by providing an “easier” path. The effects of a random environment are important in biological models such as bacterial growth or cancer initiation/progression.

## Introduction

Fixation is the replacement of an initially heterogeneous population with the offspring of just one individual. The probability of fixation and the average time that is required for a mutant to take over the population are two fundamental quantities in ecology and evolution. Both fixation probability and average fixation time have been widely studied by physicists and mathematicians for almost a century, starting with the early works by Haldane [1], Fisher [2], Wright [3], and the series of seminal papers by Kimura [4–6].

A number of stochastic models have been used to study evolution in finite populations, of which the Moran process and the Write Fisher process are perhaps the best known. The Moran process [7] assumes the existence of *N* individuals, and dynamics are modeled as a sequence of updates, such that each time one individual is chosen to be removed, and another is chosen for reproduction (thus keeping the total population size constant). *N* such elementary updates correspond to one generational update. In the Wright Fisher process (see e.g. [8]), the next generation is populated by randomly drawing (with replacement) copies of individuals from the current population.

One of the central questions that has attracted attention of researchers in the last several decades is the role of the population structure in the evolutionary dynamics. This research was pioneered by Kimura and Weiss who were the first to include spacial structure in population models [6]. Maruyama analyzed the fixation behavior of a Moran process on regular spatial structures and discovered that the fixation probability is independent of the spatial structure of the population (for example, fixation probability on regular graphs is the same as that on unstructured graphs) [9, 10]. Liberman et. al extended the analysis to arbitrary graphs (networks) [11]. They showed that some networks may act as amplifiers, and others as suppressors of selection. Namely, amplifier graphs increase (decrease) the fixation probability of advantageous (disadvantageous) and mutants; suppressors, on the contrary, decrease (increase) the fixation probability for advantageous (disadvantageous) mutants [12–14]. In [15], the role of the order of the update events (birth and death) for evolutionary dynamics in the Moran process was studied. It was discovered that for 1D and 2D spatial lattices, the fixation probabilities for birth-death and the death-birth formulations are significantly different.

Apart from fixation probability, the average fixation time is an important characteristic of birth and death processes. Much research has been devoted to studying mathematical properties of this quantity in various contexts. Frean and Baxter analyzed the mean fixation time of a mutant for two homogeneous and heterogeneous graphs [16]. They have considered four different update rules of birth-death (BD) and death-birth (BD) processes for star and complete graphs: B-FD (birth depends on fitness and death is uniform), B-DF (birth is uniform and death depends on the unfitness), D-BF (uniform death and fitness dependent birth), and DF-B (fitness dependent death and uniform birth). They have shown that the star is a suppressor in both DB processes and an amplifier in both BD cases. For further developments in the studies of the evolutionary dynamics on graphs, one can refer to the review by Shakarian et al. [17], where the authors describe the original models for evolutionary graph theory and its extensions, as well as the calculation of the fixation probability and time to fixation. Broom et al. [18] have studied the evolutionary game theory of finite structured populations with invasion process updating rules. The exact solutions are presented for the fixation probability and time for the case that mutants have fixed fitness and the case where the fitness of individuals depends on games played among the individuals, on the star, circle and complete graphs. [19] studied the importance of fixation time for the rate of evolution and showed that in star-structured populations, evolution can slow down even while selection is amplified. Hindersin and Traulsen used analytical calculations to find the fixation time of a single mutant for small graphs [20]. They showed that, interestingly, there is no obvious relation between fixation probability and time. More recently, Askari and Aghababaei-Samani introduced an exact analytical approach in order to calculate the mean time fixation of a mutant for circle and star graphs [21].

In a number of previous studies, the evolutionary properties of mutants have been investigated under the assumption that fitness values of different types were kept constant. It has been recently recognized, however, that fluctuating fitness values can have important effects on the fixation probability and time [22, 23]. In [24], the authors considered two different types of heterogeneity, a heterogeneous voter model where each voter has an intrinsic rate to change state and a partisan voter model where each voter has an innate and fixed preference for one opinion state (0 or 1). Using a mean-field approximation, they compared the time to fixation for each of these two models and studied the population-size dependency of the time to fixation (i.e. the time to ultimately reach consensus). Rivoire et al. [25] have used mathematical modeling and stochastic control theory to quantify phenotypic variation schemes, which are inherited, randomly produced, or environmentally induced, and have analyzed the adaptation towards such variations. Moreover, Melbinger and Vergassola [26] considered the effect of environmental alterations on the fitness of species. They showed that variability in the growth rates played an important role in neutral evolution and that the fixation time was reduced in the presence of time dependent environmental fluctuations. In addition, Cvijović et al. In general, studies of aspects of evolutionary dynamics related to heterogeneity have given rise to a number of interesting papers. In paper [27], a different type of population heterogenity is studied: the individuals differ by the number of connections they have with others. Paper [28] studies the fixation times in both death-birth and birth-death processes under different assumptions on the underlying network structure, the game, and fitness definition. [29] found that temporal fluctuations in environmental conditions could influence the fate of mutation and subsequently the efficiency of natural selection. They have shown that temporal fluctuations can reduce the efficiency of natural selection and increase the fixation probability of mutants, even if they are strongly deleterious on average.

In [22], we used a number of models (several versions of the Moran model and the haploid Wright-Fisher model) to examine fixation probabilities for a constant size population, where the fitness was a random function of both allelic state and spatial position. Namely, it was assumed that the fitness values of wild type cells and mutant cells were drawn from probability distributions, and were fixed for each location. Different scenarios were examined, including correlated and uncorrelated fitness values of wild type cells and mutants, and different underlying population structures (circles and complete graphs). In the case where the probability distribution of mutant and wild type cells were identical, our model of spatial heterogeneity redefined the notion of neutrality for a newly arising mutation, as such mutations fixed at a higher rate than that predicted under neutrality. In particular, it was found that the probability of mutant fixation (in the case when the mutants were initially a minority) was significantly larger than their initial fraction, and this effect increased with *N*. In other words, mutants behaved as if they were selected for, although on average their fitness values were the same as the the fitness values of wild type cells.

In the current paper we investigate the question of the timing of mutant fixation in a similar setting. Does spatial randomness of this sort influence the rate of mutant fixation, and if so, in what way? We use both analytical and numerical methods to answer this question under a variety of different assumptions on the probability distributions of wild type and mutant fitness values, and examine different biologically relevant scenarios. In particular, we investigate if spatial arrangement of cells plays a role in the timing of mutant fixation.

## Methods

### Constant population models with random fitness

Suppose a population consists of two types of individuals (or cells), A (the wild type) and B (the mutant). Consider a death-birth (DB) formulation of the Moran process. At each update, a death event is followed by a division event, where the removed individual is replaced by the offspring of one of its neighbors. While individuals are chosen for death randomly with equal probabilities, reproduction probability of each is proportional to its division rate, or fitness. Traditionally, fitness of individuals is defined by their type, such that the wild type and mutant cells have fitness values *r*_*A*_ and *r*_*B*_ respectively. The notion of neighborhood is defined by a graph connecting the individuals. For example, one may consider the complete graph, where any cell could be chosen for division to replace any eliminated cell. Another example of a graph is a circle, where each cell only has two neighbors.

In constant population processes with two sub-populations, in the absence of mutations, there are only two absorbing states: the state where all cells are wild type, and the state where all the cells are mutants. If starting from a nonzero number of mutants, the system reaches the all-mutant state, we say that the mutants have reached fixation (and otherwise we say that they have gone extinct). The expected time of fixation conditioned on the event of mutant fixation, *t*_*i*_, has been calculated for several graphs. Antal and Scheuring [30] have used an evolutionary game model to calculate fixation of strategies in finite populations. Recently, Hindersin and Traulsen [20] have obtained the fixation time for all possible connected networks with four nodes (6 graphs).

Unlike the traditional Moran process, here we consider the case where the fitness of individuals depends on their environment, in the following sense. In a system of *N* individuals, the fitness depends not only on whether an individual is wild type or mutant, but also on the location of each individual on the graph. We assume that each vertex of a graph is associated with a (fixed) wild type fitness parameter, which is generated randomly according to a given distribution, and does not change in time. Similarly, each spot is also characterized by a mutant fitness parameter. These parameters are also chosen randomly from a distribution, they characterize the fitness of mutants at different locations, and remain constant in time. The wild type and mutant fitness probability distributions may in general be the same or different, and the choice of mutant and wild type fitness values could be uncorrelated or correlated to different degrees [22]. Here, for illustration purposes, we will consider a discrete symmetric bimodal fitness distributions, such that fitness parameters are randomly selected to be 1 + *σ* or 1 − *σ*, with 0 < *σ* < 1.

In addition to the DB formulation of the Moran process described above, we also studied the birth-death (BD) formulation of the Moran process, and the haploid Wright Fisher model. In the BD Moran process, for each update, first a cell is selected for reproduction (based on the cells’ fitness values), and then a neighboring cell (excluding the reproducing cell itself) is chosen to be removed, such that all candidate cells have the same probability to be chosen. The offspring of the reproducing cell replaces the removed cell. In the haploid Wright Fisher model, each new generation of cells is created by randomly sampling (with replacement) the cell types from the current population. The probability to be picked is proportional to the cells’ fitness. Most of the ideas of this paper are illustrated by using the DB Moran model. The results obtained from the other models are similar and are presented in S1 Text.

### Calculating the mean conditional fixation time

To find the mean conditional fixation time, we use Chapman-Kolmogorov equations to find the fixation properties of a mutant. In a population of *N* individuals, there are *M* = 2^*N*^ distinct states. This can be shown by observing that in the presence of *m* mutants, 0 ≤ *m* ≤ *N*, there are *N*!/*m*!(*N* − *m*)! distinct configurations; summing those up gives the value 2^*N*^. For each fixed fitness realization, *γ*, let us denote by $T_{i\to j}^{\gamma }$ the probability of transitioning from state *i* to state *j*, 1 ≤ *i*, *j* ≤ *M*. Let us denote the absorbing state (or the set of absorbing states) of interest as *E*. In our particular case, *E* is the state where each location contains a mutant (mutant fixation). The other absorbing states comprised of the set *E*_1_ (in our case this is the state of all wild type cells, that is, mutant extinction). Following [30], denote by $\rho_{i}^{\gamma }\left(t\right)$ the probability to get absorbed in state *E* starting from state *i* after *t* steps, under fitness configuration *γ*. The total probability of absorption in *E* is then given by

$$\begin{array}{c} \rho_{i}^{\gamma }=\sum_{t=0}^{\infty }\rho_{i}^{\gamma }\left(t\right). \end{array}$$

We have for this quantity,

$$\begin{array}{c} \rho_{i}^{\gamma }=\sum_{j}T_{i\to j}^{\gamma }\rho_{j}^{\gamma },\;i\notin E,E_{1}, \end{array}$$ (1)

where the summation in *j* goes over all the states, and

$$\begin{array}{c} \rho_{E}^{\gamma }=1,\;\rho_{E_{1}}^{\gamma }=0. \end{array}$$ (2)

This is a linear system of *M* − 2 equations for $\rho_{i}^{\gamma }$ (the Chapman-Kolmogorov equation [31]). Next, let us denote by $t_{i}^{\gamma }$ the mean conditional time it takes to get absorbed in state *E* starting from state *i* under configuration *γ*, given that absorption happens:

$$\begin{array}{c} t_{i}^{\gamma }=\frac{\sum_{t=0}^{\infty }t\rho_{i}^{\gamma }\left(t\right)}{\sum_{t=0}^{\infty }\rho_{i}^{\gamma }\left(t\right)}=\frac{\sum_{t=0}^{\infty }t\rho_{i}^{\gamma }\left(t\right)}{\rho_{i}^{\gamma }}, \end{array}$$

and further denote

$$\begin{array}{c} \tau_{i}^{\gamma }=t_{i}^{\gamma }\rho_{i}^{\gamma }. \end{array}$$

We have

$$\begin{array}{c} \rho_{i}^{\gamma }\left(t\right)=\sum_{j}T_{i\to j}^{\gamma }\rho_{j}^{\gamma }\left(t-1\right). \end{array}$$ (3)

Changing the summation index, we obtain

$$\begin{array}{c} \sum_{t=0}^{\infty }t\rho_{j}^{\gamma }\left(t-1\right)=\sum_{t=0}^{\infty }\left(t+1\right)\rho_{j}^{\gamma }\left(t\right)=\tau_{j}^{\gamma }+\rho_{j}^{\gamma }. \end{array}$$

Multiplying eq (3) by *t* and summing up from 0 to infinity, we obtain

$$\begin{array}{c} \tau_{i}^{\gamma }=\sum_{j}T_{i\to j}^{\gamma }\tau_{j}^{\gamma }+\sum_{j}T_{i\to j}^{\gamma }\rho_{j}^{\gamma }, \end{array}$$

or equivalently,

$$\begin{array}{c} \tau_{i}^{\gamma }=\sum_{j}T_{i\to j}^{\gamma }\tau_{j}^{\gamma }+\rho_{i}^{\gamma },\;i\notin E,E_{1}, \end{array}$$ (4)

where the summation in *j* goes over all the states, and

$$\begin{array}{c} \tau_{E}^{\gamma }=0,\;\tau_{E_{1}}^{\gamma }=0. \end{array}$$ (5)

Eqs (1) and (4) comprise a closed system that can be solved for $\rho_{i}^{\gamma }$ and $\tau_{i}^{\gamma }$ for all *i* ∉ *E*, *E*_1_. Then, the conditional mean time of absorption under configuration *γ* is given by

$$\begin{array}{c} t_{i}^{\gamma }=\frac{\tau_{i}^{\gamma }}{\rho_{i}^{\gamma }}. \end{array}$$

We are interested in the expectation of this quantity over all realizations of the fitness realization, *γ*:

$$\begin{array}{c} E_{\gamma }\left[t_{i}^{\gamma }\right]=E_{\gamma }[\frac{\tau_{i}^{\gamma }}{\rho_{i}^{\gamma }}]. \end{array}$$ (6)

**Fig 1:**
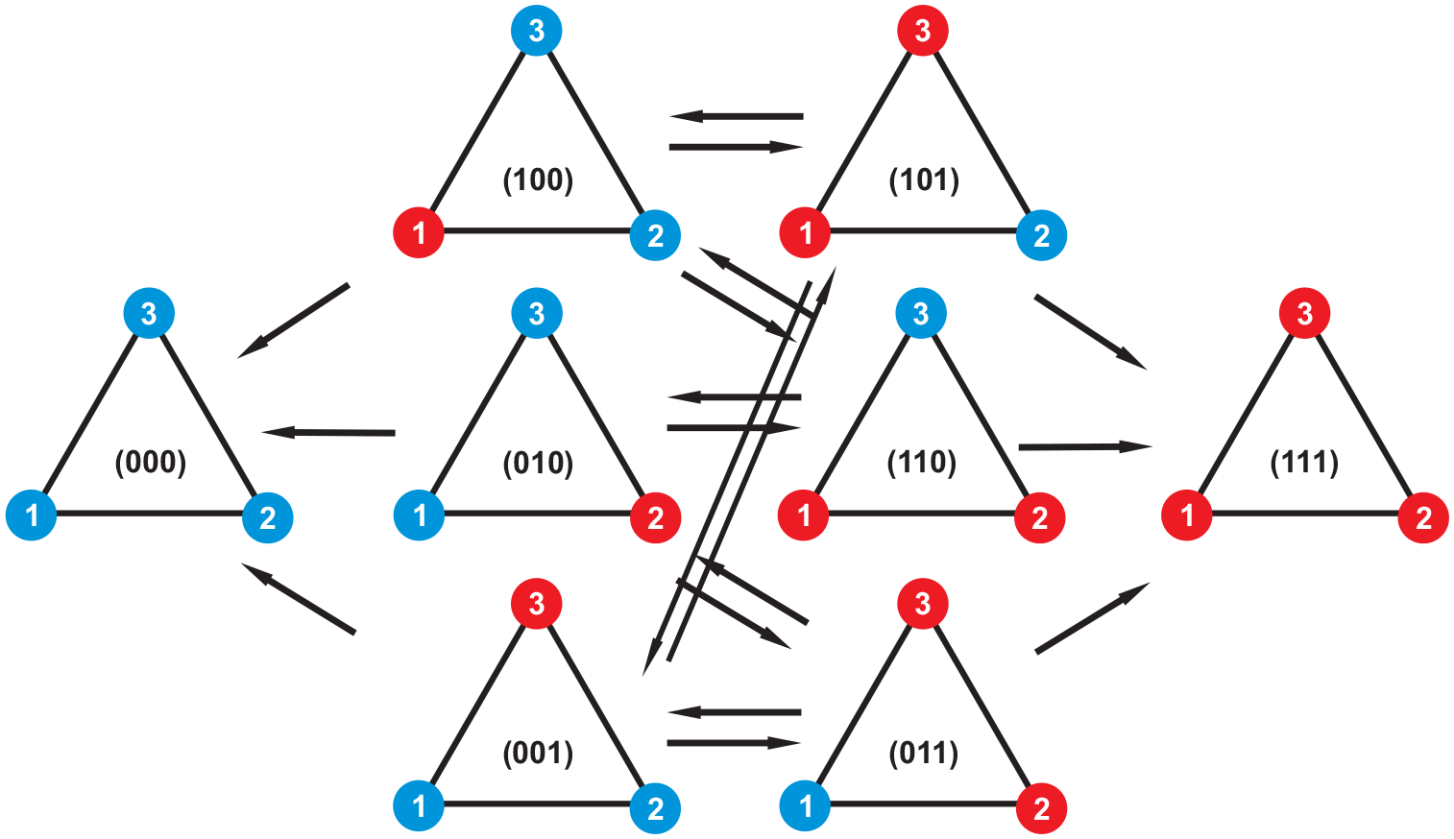
The eight possible configurations on a graph of size *N* = 3. Red nodes show mutants and blue nodes indicate wild types. The process starts with one of the states containing a mutant and continuous until it reaches one of the absorbing states with three mutants or three wild-types. The arrows show possible transitions between states of the Markov chain at each time step.

As an example, we apply this theory to the circle graph in the context of the death-birth Moran process. For illustration purposes, we use the population size *N* = 3 (note that in this case, the circle and the complete graph are the same). Denote the states of the Markov chain by vector (*n*_1_, *n*_2_, *n*_3_), where *n*_*i*_ = 1 if the site is occupied by a mutant and *n*_*i*_ = 0 otherwise. There are two absorbing states: (000), the state occupied with all wild-type cells, and (111), the state filled entirely with mutants. The states (100), (010) and (001) are the states of Markov chain with one mutant and (101), (110) and (011) are the states with two mutants (Fig 1). We use the notation (*a*, *b*, *c*) to denote wild type fitness values at locations (1, 2, 3); the mutant fitness values at these locations are denoted by $\left(\tilde{a},\tilde{b},\tilde{c}\right)$. For each fitness configuration, the probability of reaching the state *E* (the state of all mutant cells) starting from state (*n*_1_*n*_2_*n*_3_) is defined by $\rho_{n_{1}n_{2}n_{3}}$. Using formulas (1) and (2), one can write Chapman-Kolmogorov equations for the fixation probability under a fixed set of fitness values,

$$\begin{array}{ccc} 3\rho_{100} & = & \frac{\tilde{a}}{\tilde{a}+b}\rho_{101}+\frac{\tilde{a}}{\tilde{a}+c}\rho_{110}+\left(\frac{b}{\tilde{a}+b}+\frac{c}{\tilde{a}+c}\right)\rho_{100},\\ 3\rho_{010} & = & \frac{\tilde{b}}{\tilde{b}+c}\rho_{110}+\frac{\tilde{b}}{\tilde{b}+a}\rho_{011}+\left(\frac{c}{\tilde{b}+c}+\frac{a}{\tilde{b}+a}\right)\rho_{010},\\ 3\rho_{001} & = & \frac{\tilde{c}}{\tilde{c}+a}\rho_{011}+\frac{\tilde{c}}{\tilde{c}+b}\rho_{101}+\left(\frac{a}{\tilde{c}+a}+\frac{b}{\tilde{c}+b}\right)\rho_{001},\\ 3\rho_{101} & = & \frac{b}{\tilde{a}+b}\rho_{100}+\frac{b}{\tilde{c}+b}\rho_{001}+1+\left(\frac{\tilde{a}}{\tilde{a}+b}+\frac{\tilde{c}}{\tilde{c}+b}\right)\rho_{101},\\ 3\rho_{110} & = & \frac{c}{\tilde{a}+c}\rho_{100}+\frac{c}{\tilde{b}+c}\rho_{010}+1+\left(\frac{\tilde{a}}{\tilde{a}+c}+\frac{\tilde{b}}{\tilde{b}+c}\right)\rho_{110},\\ 3\rho_{011} & = & \frac{a}{\tilde{b}+a}\rho_{010}+\frac{a}{\tilde{c}+a}\rho_{001}+1+\left(\frac{\tilde{b}}{\tilde{b}+a}+\frac{\tilde{c}}{\tilde{c}+a}\right)\rho_{011}. \end{array}$$ (7)

Denote by ${\tilde{\rho }}_{m}$ the fixation probability starting with *m* mutants, averaged over all realizations of the fitness value sets. For *N* = 3, if the mutant and wild type fitness values are generated from the same distribution, the average fixation probability is a constant independent of the probability distribution [22]:

$$\begin{array}{c} {\tilde{\rho }}_{1}=\frac{1}{3},\;{\tilde{\rho }}_{2}=\frac{2}{3}, \end{array}$$ (8)

which coincides with the result *ρ*_*m*_ = *m*/*N* for neutral mutants, in the absence of randomness. Note that for larger values of *N* (namely, all *N* > 3), the mutant fixation probability is no longer *m*/*N*, and it depends on the distribution. In particular, for minority mutants (that is, for *m* < *N*/2), the probability of fixation increases with the variance of the underlying distribution [22].

Denoting by $t_{n_{1}n_{2}n_{3}}=\tau_{n_{1}n_{2}n_{3}}/\rho_{n_{1}n_{2}n_{3}}$ the mean fixation time needed for going from state (*n*_1_*n*_2_*n*_3_) to state (111), under a fixed fitness realization, we obtain the following six linear Chapman-Kolmogorov equations (see eqs (4) and (5)):

$$\begin{array}{ccc} 3\rho_{100} & = & -\frac{\tilde{a}}{\tilde{a}+b}\tau_{101}-\frac{\tilde{a}}{\tilde{a}+c}\tau_{110}+\left(1+\frac{\tilde{a}}{\tilde{a}+b}+\frac{\tilde{a}}{\tilde{a}+c}\right)\tau_{100},\\ 3\rho_{010} & = & -\frac{\tilde{b}}{\tilde{b}+c}\tau_{110}-\frac{\tilde{b}}{\tilde{b}+a}\tau_{011}+\left(1+\frac{\tilde{b}}{\tilde{b}+c}+\frac{\tilde{b}}{\tilde{b}+a}\right)\tau_{010},\\ 3\rho_{001} & = & -\frac{\tilde{c}}{\tilde{c}+a}\tau_{011}-\frac{\tilde{c}}{\tilde{c}+b}\tau_{101}+\left(1+\frac{\tilde{c}}{\tilde{c}+a}+\frac{\tilde{c}}{\tilde{c}+b}\right)\tau_{001},\\ 3\rho_{101} & = & -\frac{b}{\tilde{a}+b}\tau_{100}-\frac{b}{\tilde{c}+b}\tau_{001}+\left(1+\frac{b}{\tilde{a}+b}+\frac{b}{\tilde{c}+b}\right)\tau_{101},\\ 3\rho_{110} & = & -\frac{c}{\tilde{a}+c}\tau_{100}-\frac{c}{\tilde{b}+c}\tau_{010}+\left(1+\frac{c}{\tilde{a}+c}+\frac{c}{\tilde{b}+c}\right)\tau_{110},\\ 3\rho_{011} & = & -\frac{a}{\tilde{b}+a}\tau_{010}-\frac{a}{\tilde{c}+a}\tau_{001}+\left(1+\frac{a}{\tilde{b}+a}+\frac{a}{\tilde{c}+a}\right)\tau_{011}. \end{array}$$ (9)

The mean conditional fixation time averaged over all realizations of the fitness landscapes is then obtained from eq (6). For *N* = 3, we can solve the above equations analytically, and then the mean conditional fixation time (averaged over all realizations of the fitness values) is obtained from eq (6). We have written a *Mathematica* code that generates the set of equations for any *N* (the equations for *N* = 4 are presented in S1 Text). However, this approach is only practical for small values of *N* (due to the large number of equations and configurations). For larger networks, we use the canonical matrix method [32] (see S1 Text) or stochastic simulations to find the fixation probability and the mean conditional fixation time.

### Stochastic simulations

The equations described above only have practical applicability for relatively small networks. As *N* increases, the number of equations grows as 2^*N*^ − 2 for the complete graph and *N*(*N* − 1) for the circle. Instead of solving the algebraic equations, stochastic numerical simulations have to be implemented.

In the numerical simulations, we consider the population on a graph, where each vertex is an individual (wild type or mutant). For each simulation, we generate wild type fitness values and mutant fitness values from their respective probability distributions. These values are associated with their vertices on the graph and are kept constant until the end of the simulation. Since the fitness values are randomly selected from a bimodal distribution (1 + *σ* and 1 − *σ*) for each individual at every node of the graph, there will be 2^2*N*^ fitness configurations.

We start with an initial condition of one mutant (type *B*) and *N* − 1 wild types (type *A*). At each time step, as long as the mutant population has not yet become fixated or gone extinct, one individual is randomly removed and one of its neighbors is chosen for division with a probability proportional to its fitnesses. The simulation is stopped when the mutants become extinct or reach fixation. If mutant fixation is reached, we record the number of updates until fixation; this gives the time to fixation for a particular (successful) run. This process is repeated a number of times for each configuration. The mean conditional time for *each configuration* is the sum of all individual fixation times divided by the number of successful samples (that is, the number of runs where mutant fixation was observed). The overall mean conditional fixation time is the average of the mean conditional fixation times over all possible configurations.

A computational difficulty with this approach arises from the fact that for some fitness configurations, the probability of mutant fixation is very low. For such configurations, after running the simulation a fixed number of times, it may happen that fixation never occurs, in which case the configuration will not contribute into the calculated mean conditional fixation time. The configurations with low fixation probabilities are less likely to be fixed, which may skew the numerical results. In order to avoid this problem, we executed over 10^6^ independent realizations for each configuration. As a result, the simulation is very costly.

For larger networks, instead of the exhaustive calculation described above, we used a sampling method similar to the method that was implemented in [22]. Since listing all the configurations becomes computationally impossible, we only looked at a subset of possible realizations of random fitness values. For each such realization, we ran simulations starting from one mutant cell, until the mutants reached fixation. At this point, the time it took to fixate was recorded, and we moved on to the next randomly chosen configuration. The mean conditional fixation time was then approximated as an average over fixation times obtained for these realizations.

## Results

The main results are presented for the DB Moran process. The BD Moran and the Wright Fisher model show similar trends and are presented in S1 Text.

### Small circles: Complex parameter dependencies

We use Chapman-Kolmogorov equations presented above to calculate the mean conditional fixation time. We start by examining the case of circular networks.

#### Dependence on the standard deviation

**Fig 2:**
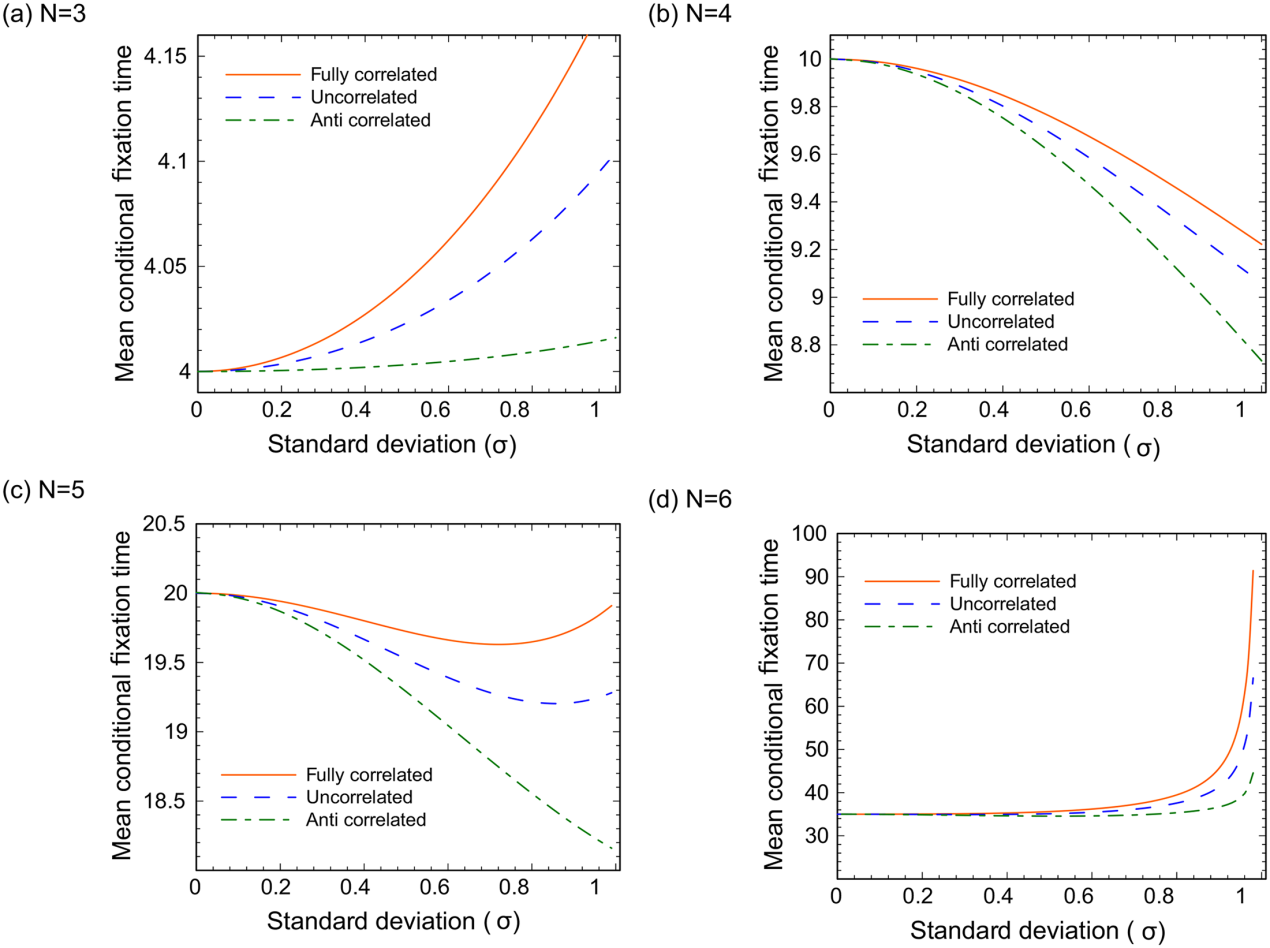
Circular graphs: The dependence of the mean conditional mutant fixation time (starting with one mutant) on standard deviation. (a) *N* = 3. (b) *N* = 4. (c) *N* = 5. (d) *N* = 6. Results for three cases are presented: mutant and wild type fitness values are fully correlated, uncorrelated, and anti-correlated.

Suppose that fitness values for mutants and wild types are drawn from the same distribution, and use the example of a two-valued distribution function, where values 1 − *σ* and 1 + *σ* are equally likely. In Fig 2 we perform calculations for *N* = 3 through *N* = 6 (panels (a)-(d)), and plot the dependence of mean conditional fixation time on the standard deviation, *σ*, which can be interpreted as the amount of randomness in the system. We start the discussion with the case where fitness values of wild types and mutants are chosen independently from the same distribution (blue lines in Fig 2). We notice immediately that the mean conditional time to fixation depends on the amount of randomness, even for *N* = 3. This is in contrast with the mean probability of fixation for *N* = 3 (see eq (8)), which is independent of *σ* for the DB Moran process.

Interestingly, the type of dependence changes with system size, *N*. The following patterns are observed. At *σ* = 0, the non-random values 4, 10, 20, and 35 are obtained for *N* = 3, 4, 5, 6 respectively. For nonzero values of *σ*, the probability distribution of fixation times are presented in S1 Text. As expected, for larger values of *N* fixation takes longer, and the probability distribution of the fixation times becomes larger. Finally, we notice in Fig 2 that as the value of *σ* increases, the mean conditional time to fixation increases for *N* = 3, decreases for *N* = 4, then it is nonmonotonic for *N* = 5, and it increases rapidly for *N* = 6. As will be shown below, for larger values of *N*, the mean conditional fixation time is an increasing function of *σ*.

#### The effect of correlations

In Fig 2, the effect of correlations between mutant and wild type fitness values is also studied. As mentioned above, the blue lines show the case where the wild type and mutant fitness values are assigned independently. The green lines represent the case of anti-correlated fitness values (such that the mutant fitness at a given location is 1 − *σ* if the wild type fitness is 1 + *σ*, and vice versa). Finally, the orange lines correspond to fully correlated fitness values, where mutants have the same fitness values as the wild types at each location. We observe that the mean conditional fixation time is always the largest for the fully correlated case and the smallest for anti-correlated case.

Interestingly, the effect of randomness does not disappear for the fully correlated case, and in fact for *N* = 3 and *N* = 6 it is the largest for the fully correlated case. It was shown in [22] that the probability of mutant fixation in the fully correlated case equals 1/*N* for all *N* with *N* ≥ 3. With time to fixation, we can see that randomness plays a role even if the mutants behave exactly as the wild types.

#### The role of skewness

Next we investigate the effect of skewness of the fitness probability distributions on fixation times. Again, we illustrate the results by using a two-valued fitness probability distribution with values *x*_1_ and *x*_2_. We assume that *p*(*x*_1_) = *p*, *p*(*x*_2_) = 1 − *p*, where skewness is simply

$$\begin{array}{c} S=p_{1}{\left(x_{1}-\mu \right)}^{3}+p_{2}{\left(x_{2}-\mu \right)}^{3},\;\mu =p_{1}x_{1}+p_{2}x_{2}. \end{array}$$

Quantities *p*, *x*_1_, *x*_2_ can be expressed in terms of the mean *μ*, variance *σ*^2^, and skewness *S*, as

$$\begin{array}{c} x_{1,2}=\frac{2\mu \sigma^{2}+S\mp \sqrt{4\sigma^{6}+S^{2}}}{2\sigma^{2}},\;p=\frac{1}{2}\;(1+\frac{S}{\sqrt{4\sigma^{6}+S^{2}}}). \end{array}$$ (10)

**Fig 3:**
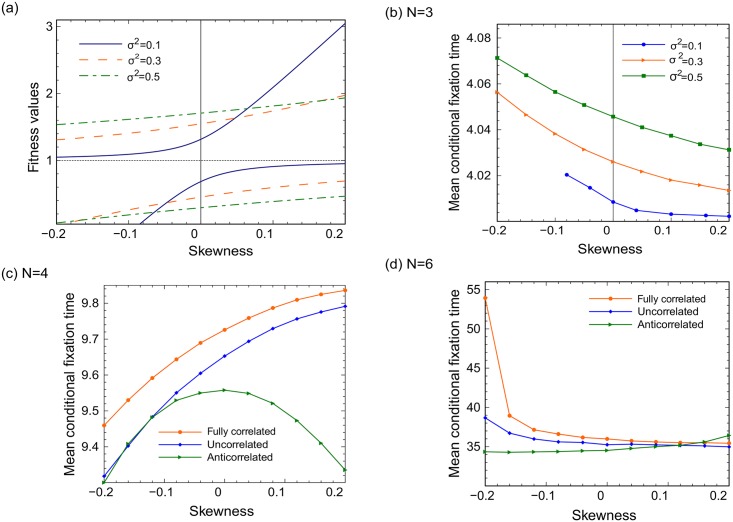
Circular graphs: The effect of skewness of the fitness probability distribution. (a) The concept of skewness is illustrated for the two-value distribution. For fixed mean (*μ* = 1) and three values of variance, the two possible fitness values, *x*_1_ and *x*_2_, eq (10), are plotted as functions of the skewness. (b) For the same three values of variance, the mean conditional fixation time in the *N* = 3 system is plotted as a function of skewness. The effect of correlations is added in (c) and (d), where the mean conditional fixation time is plotted as a function of skewness for (c) *N* = 4 and (d) *N* = 6, with *μ* = 1, *σ*^2^ = 0.3, and three correlation conditions: correlated, uncorrelated, and anti-correlated fitness values.

For zero skewness, *x*_1_ and *x*_2_ are equidistant from the mean (Fig 3(a)), while for positive skewness, *x*_1_ is near the mean and *x*_2_ is large (and unlikely), and for negative skewness *x*_2_ is near the mean and *x*_1_ is small (and unlikely). We assume that both wild types and mutants have identical fitness distribution functions, and calculate the mean conditional fixation times for such distributions. Fig 3(b) presents results for *N* = 3. It turns out that in this system, mean conditional fixation time in a decreasing function of skewness. It follows that the effect of randomness (delay in fixation) is the largest for negative skewness values. Panels Fig 3(c) and 3(d) show the dependence of fixation time on skewness in the case of *N* = 4 and *N* = 6. Superficially, the result for *N* = 4 is in some sense the opposite of the *N* = 3 and *N* = 6 cases: the fixation time is an increasing function of skewness for *N* = 4. One trend remains unchanged for uncorrelated and anti-correlated cases: as in the *N* = 3 case, the effect of randomness (whether it is acceleration of fixation, as observed for *N* = 4, or deceleration of fixation, as observed for *N* = 3 and *N* = 6 in the uncorrelated and fully correlated cases) is felt the most for negative skewness values. A more detailed study of the effect of skewness is presented in S1 Text.

### Small complete graphs: Mean conditional time decreases with randomness

**Fig 4:**
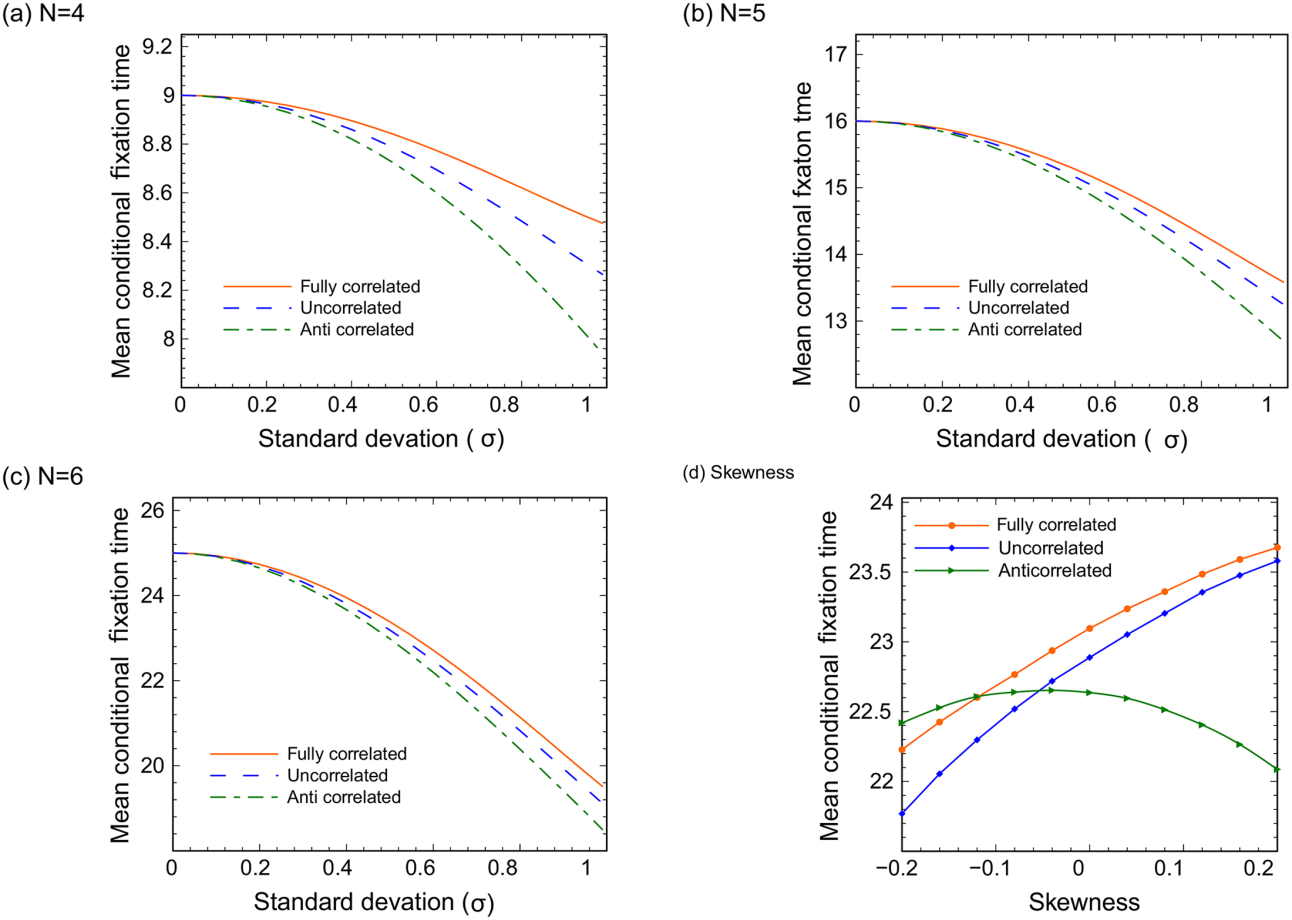
Small complete graphs: The effects of correlation and skewness. (a-c): The conditional mean mutant fixation time is plotted as a function of the standard deviation, for (a) *N* = 4, (b) *N* = 5, (c) *N* = 6. The effect of skewness of the fitness probability distribution for *N* = 6 is shown in (d), where the mean conditional fixation time is plotted as a function of skewness, with *μ* = 1, *σ*^2^ = 0.3. In all panels, results for three cases are presented: mutant and wild type fitness values are fully correlated, uncorrelated, and anti-correlated.

So far, all the calculations were performed for small circular networks. Next, we turn to complete graphs. In Fig 4 we perform calculations on a complete graph for *N* = 4 through *N* = 6 (panel (a)-(c)) and show the dependence of mean conditional time on *σ*. In contrast with the results for the circular graphs, the type of dependence does not change with system size, *N*. As expected, for *σ* = 0 the non-random values 9, 16, 25 are obtained. As the value of *σ* increases, the mean time to fixation decreases. As will be shown below, for larger values of N, the mean conditional time is also a decreasing function of *σ*. This result is quite general. In S1 Text, we extended our calculations for the mean conditional fixation time on complete graphs to the BD formulation of the Moran process and to the (haploid) Wright Fisher model [22]. In these cases, the mean conditional fixation time is also a decreasing function of *σ*.

The effect of correlations between mutant and wild-type fitness values is studied in Fig 4(a)–4(c). As for circular graphs, the mean conditional fixation time is always the largest for the fully correlated case and the smallest for the anti-correlated case. Fig 4(d) shows the dependence of fixation time on skewness in the case of *N* = 6 (other values of *N* show similar trends); the behavior is again qualitatively similar to that of the circular graphs. Finally, in S1 Text we studied the probability distribution of the time to fixation for nonzero *σ*. As in the case of small circles, the distribution of fixation times becomes wider with *N*.

### Fitness probability distributions of mutants and wild types differ

So far we have considered the scenario where the probability distributions of the wild type and mutant fitness values were the same. Here we allow them to be different, and study two interesting cases: (a,b) only one of the two types has random fitness values, while the other is deterministic, keeping the same mean fitness; (c,d) both types are random, but one of the types is advantageous.

**Fig 5:**
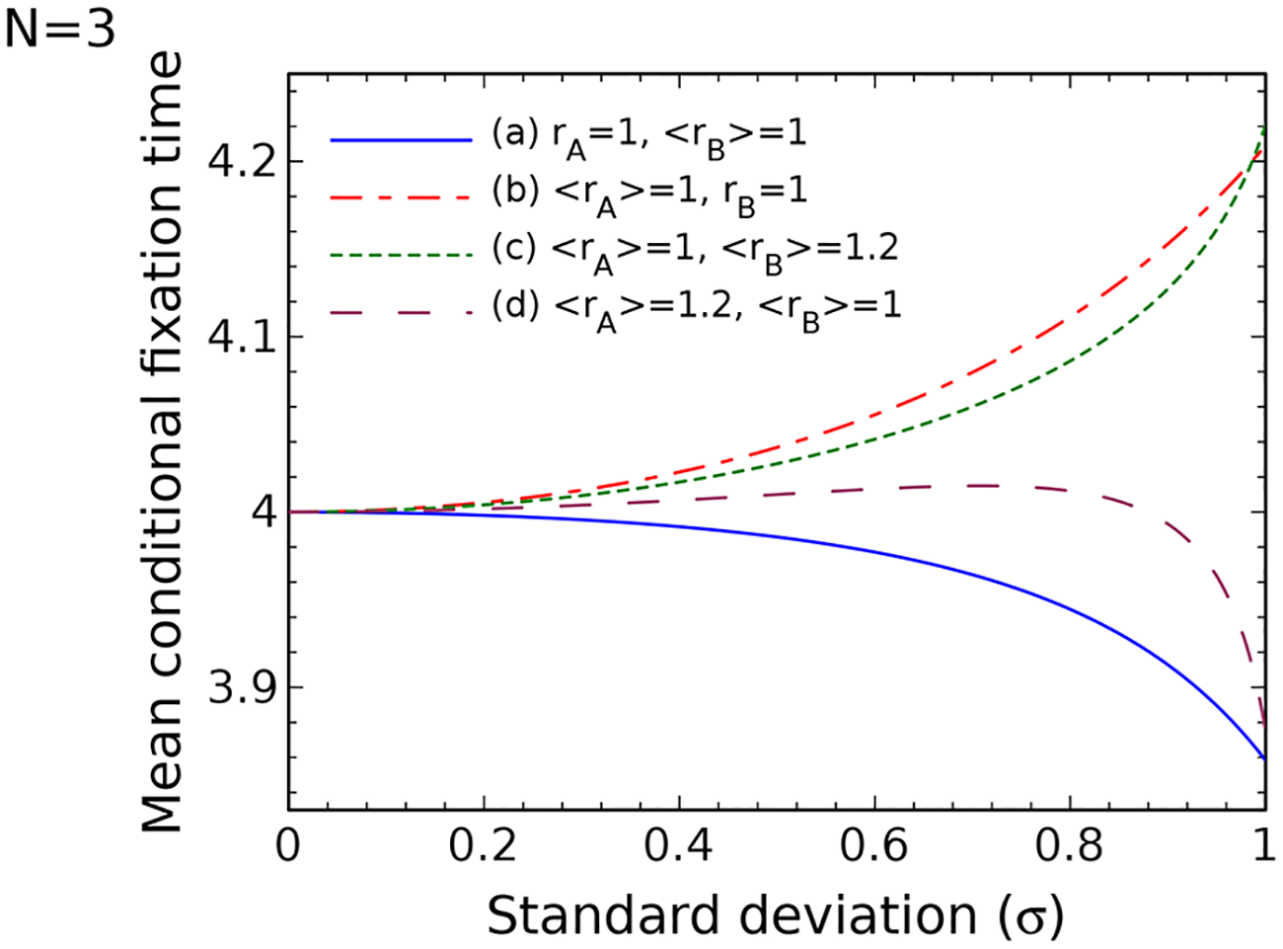
Different fitness distributions for wild types and mutants: The mean conditional fixation time on a circle/complete graph with population size *N* = 3. (a) The mutants have random fitness with average one and the wild-type individuals have a constant fitness 1. (b) The mutants have fixed fitness 1 and the wild types have a random fitness with average one. (c) The mutants have random fitness with average 1.2 and wild types have random fitness with average one. (d) The wild types have random fitness with average 1.2 and mutants have random fitness with average one.

Fig 5 explores the above cases for the *N* = 3 network. We can see large differences in the behavior of fixation times, depending on which type is random and which type is advantageous. For case (a) where only the fitness of mutant individuals is changing and case (d) where the wild-types are advantageous, the fixation time is a decreasing function of the standard deviation *σ*. For case (b), where the mutant cells are assumed to have fixed fitness 1 and the wild-types have a random fitness with average 1, and case (c) where the mutants are advantageous, the fixation time is an increasing function of standard deviation. Interestingly, these results do not generalize for larger values on *N*, and the complexity disappears as *N* increases. What we find is that all the four situations exhibit similar dependencies, and the main factor determining the behavior is the type of network.

**Fig 6:**
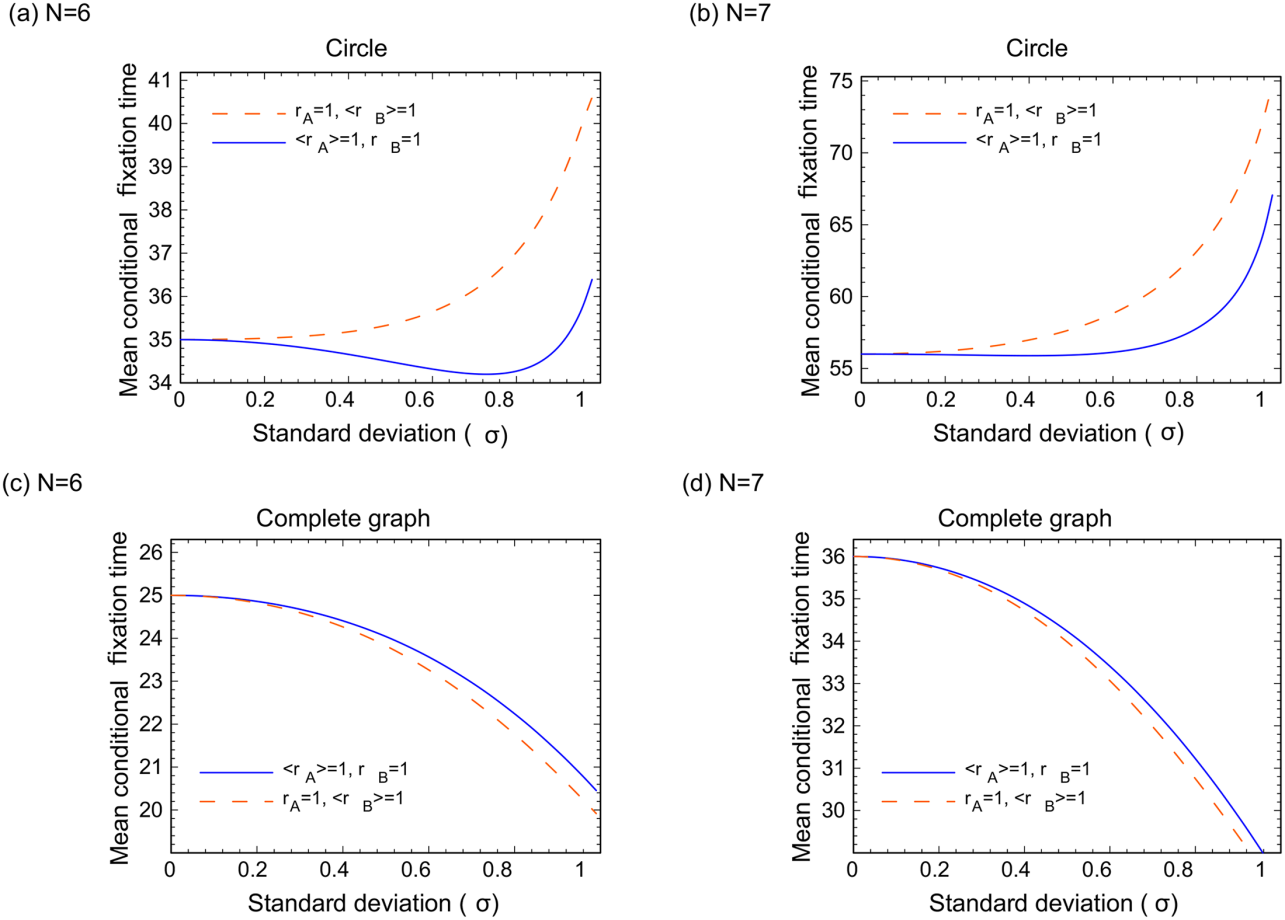
The effect of randomness on the fixation time in the case where only one of the types has random fitness values. (a,b) Circular graph and (c,d) Complete graph (*N* = 6 and *N* = 7). The orange lines correspond to constant fitness of the mutant, and the blue lines to constant fitness of the wild-types. The vertical axis is the mean conditional fixation time, and the horizontal axis is *σ*.

**Fig 7:**
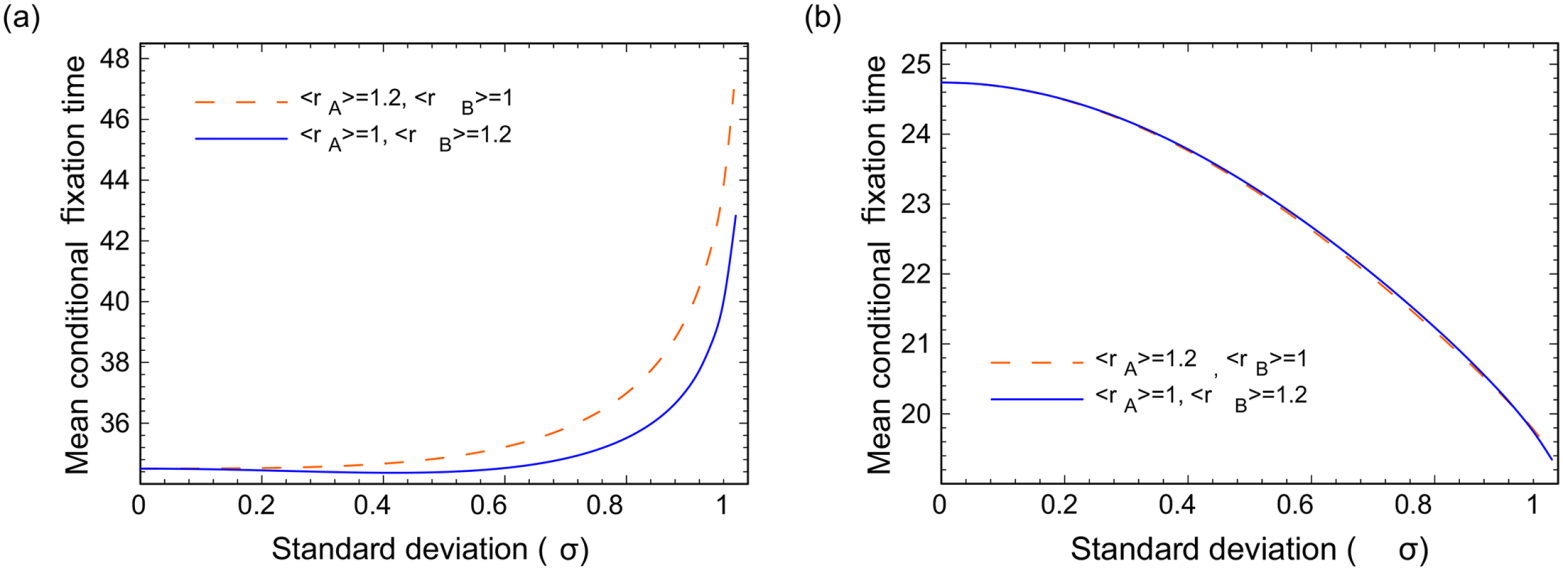
The effect of randomness on the fixation time for advantageous and disadvantageous mutants; *N* = 6. (a) Circular graph, (b) complete graph (note that the two lines overlap). The vertical axis is the mean conditional fixation time, and the horizontal axis is *σ*.

In Fig 6, we increase the population size to *N* = 6 and *N* = 7 and study cases where only one of the species (mutants or wild types) have random fitness, whereas the other species has a fixed fitness value, with both fitness values having the same mean. Panels (c) and (d) correspond to complete graphs, and it is clear that randomness accelerates fixation. Panels (a) and (b) show the results for circular graphs. While some non-monotonicity is present for the case of random wild types and deterministic mutants, it disappears for larger *N*, and the general result for circles is that randomness delays fixation. Fig 7 studies the case where either wild types or mutants are advantageous, while both cell types have random fitness. Again, randomness delays fixation for circular graphs (a) and accelerates it for complete graphs (b).

These results are consistent with the rest of the findings for circular networks and complete graphs. We delay an intuitive explanation for this phenomenon until the next section.

### Larger networks

To investigate the effect of random environment on the mean fixation time for larger networks, we turn to stochastic simulations. Again, we consider the complete graph and circle arrangement with different population sizes. The simulated results are given for the DB Moran process in the case where the fitnesses of both kinds of individuals are selected from random (binomial) distribution with average one, i.e. 1 + *σ* or 1 − *σ*.

**Fig 8:**
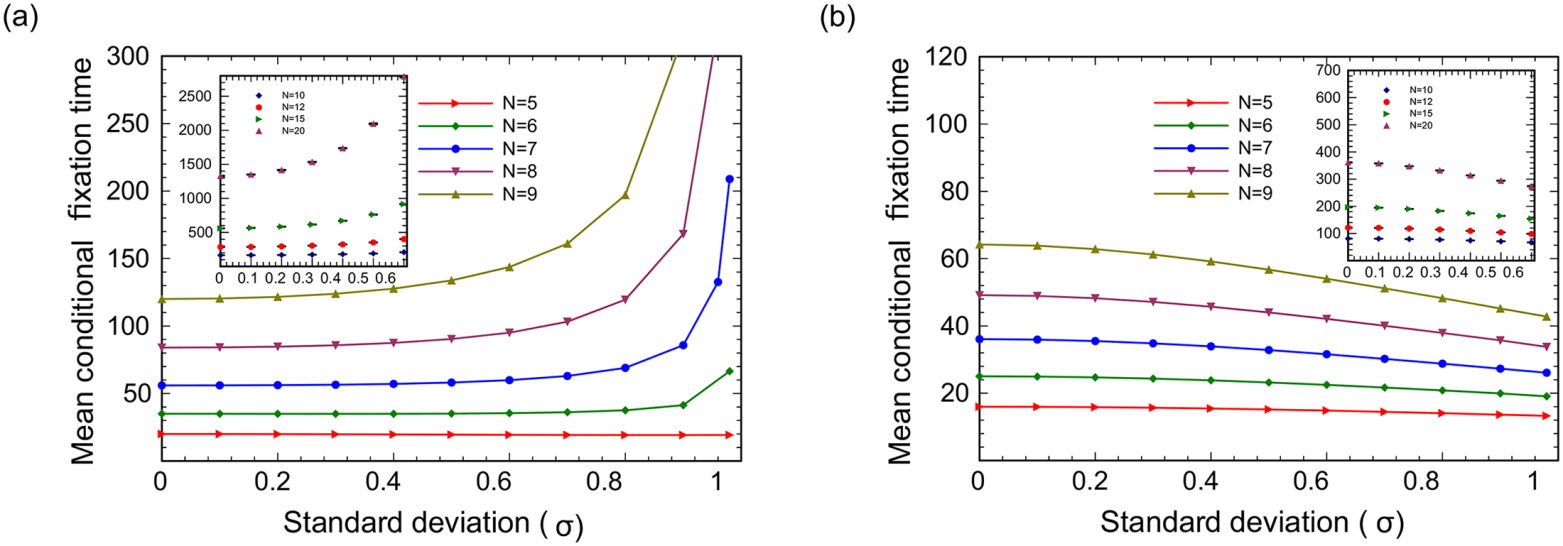
The conditional fixation time on two different networks (a) circles and (b) complete graphs for population sizes *N* = 5, 6, ⋯, 9 (exact stochastic simulations, dots, and analytical results obtained by the matrix method, lines) and *N* = 10, 12, 15, 20 (stochastic sampling simulations). For each value of *σ*, 10^6^ random configurations were used, and the calculations repeated 6 times, to obtain the standard deviation, presented as error bars.

First, we investigate the impact of random fitness on the mean conditional fixation time of a mutant for circle and complete graph with different population sizes (Fig 8). We observe that, as expected, the larger the population size *N*, the larger the fixation time of the mutants. Further, for equal values of *N*, the fixation happens faster on a complete graph than on a circle. This is also expected, as there are more pathways for mutants to spread on a complete graph, compared to a circle where a (one-dimensional) mutant patch can only grow through its two boundaries.

Next, we explore the dependence of the mean fixation time on randomness. Recall that in the case of circles (see Fig 2), for *N* = 3, the mean conditional fixation time increased with *σ*, it decreased with *σ* for *N* = 4, was non-monotonic for *N* = 5, and increased again with *N* = 6. It turns out that the trend observed for *N* = 6 persists for larger values on *N*, see Fig 8(a), where we can see that the mean conditional fixation time increases with standard deviation.

Interestingly, the result for the complete graph is very different (Fig 8(b)). There, the mean conditional fixation time is a decreasing function of the standard deviation for all population sizes. The explanation for this phenomenon is quite intuitive. As mentioned above, on a circle, the mutant population spreads out from a single mutant as a connected patch. This patch must expand to the whole circle to reach fixation, and the presence of a fitness “dead zone” (a sequence of several consecutive low fitness values for the mutants on the random fitness landscape) serves as a hurdle that can significantly increase fixation time, as there is no way around those dead zones. On the other hand, a complete graph allows many “paths” to fixation, because every spot is everyone’s neighbor, and the presence of several low fitness spots does not preclude the mutants from spreading in the same way as it does in a 1D geometry. Moreover, for a fully connected graph, the presence of randomness actually creates opportunities, increasing the likelihood of “lucky” paths to fixation, where several “neighboring” spots have an elevated mutant fitness. This explains a decrease in the expected fixation time as randomness on a complete graph increases, Fig 8(b).

We note that for small circles, the dependence of the mean conditional fixation time on randomness is less straightforward, because for very small networks the difference between the number of pathways to fixation on a circle and on a complete graph is not as drastic as it is for larger *N*.

This explanation of the effect of randomness on fixation time holds also for the scenarios where the fitness probability distributions are different for mutants and wild type cells. The following scenarios of interest were studied in the previous section. (i) The expected fitness value is the same for the wild types and the mutants, but either the wild type or mutant fitness values are constant (non-random and equal to their expectation), while the other type’s fitness values have a nonzero variance. (ii) Both types have random fitness values, but the mean fitness of mutants is larger or smaller than that of the wild types. In all these cases, it was observed that for sufficiently large values of *N*, the mean conditional mutant fixation time is an increasing function of randomness for circles and a decreasing function of randomness for complete graphs.

To understand the drastic changes in the behavior for small networks (Fig 5) and larger networks (Figs 6 and 7), let us first turn to Figs 6(b) and 7(a), which describe larger circles and contain all 4 cases (a,b,c,d) listed in Fig 5. They all show an increase in the fixation time as randomness increases, which coincides with our prediction for circles. Next, consider Figs 6(a) and 7(b), both of which describe larger complete graph networks. Again, these two figures contain all 4 cases (a,b,c,d), and they all show a decrease in the fixation time as randomness increases. This is the result that we predict for complete graphs. The way one could intuitively understand the behavior of the *N* = 3 network of Fig 5 is to remember that this network is simultaneously a circle and a complete graph. It turns out that when it comes to deterministic or advantageous mutants, the *N* = 3 network behaves as if it was a circle. In the case of deterministic or advantageous wild types, it behaves as if it were a complete graph. For larger networks this “confusion” disappears and the results are consistent with our intuition.

**Fig 9:**
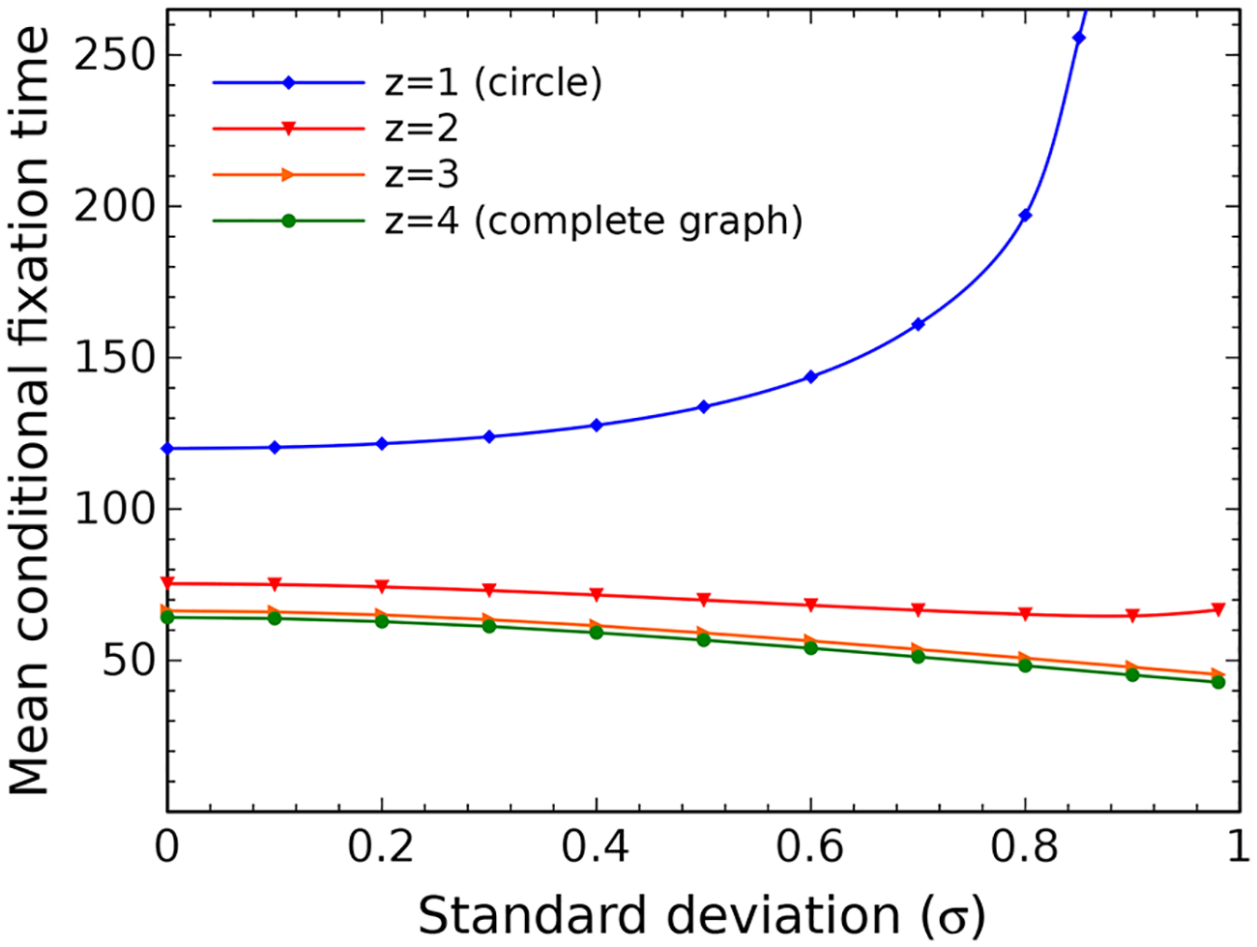
The average conditional fixation time for a mutant with random fitness on a graph with *N* = 9 nodes and different numbers of neighbors (different degrees *z*). Lines represent the analytical results and each data point is averaged over 10^6^ independent realizations.

The ideas presented here are illustrated further when we compare the results of mean fixation time for regular graphs with different degrees (see Fig 9). Define the variable *z* as the half of the number of neighbors for each node. For a regular graph with size *N* = 9, the parameter *z* = 1 corresponds to the circle structure with the nearest neighborhood for each node, *z* = 2 corresponds to the circle with the second nearest neighborhood and so on, until *z* = 4 which corresponds to the complete graph. Increasing the value of *z*, the mean fixation time decreases, and as a function of *σ*, it also switches from an increasing to a decreasing function.

We have also studied the behavior of mutant fixation behavior as a function of the initial number of mutants. We have calculated the unconditional absorption time, which is the expected time to get into either of the two absorbing states (all mutants or all wild-type cells). The results are presented in S1 Text and are consistent with the rest of the findings: unconditional mean absorption time grows with randomness for circles and decreases for complete graphs.

## Discussion

In summary, we have studied several constant population models (two formulations of the Moran model and the Wright Fisher model), where the fitness values of cells depend not only on their types (mutant or wild type) but also on their spatial locations, representing environmental factors. Fitness values of mutants and wild type cells at different locations are drawn from fixed probability distributions and remain constant in time. We ask how mutant (conditional) fixation times are influenced by this type of environmental randomness.

Before we summarize the results, we want to emphasize the applications that motivated this study and to show that our approach is rooted in real biological problems. Let us think of a spatially distributed population, say, a number of plants in an expanse of soil. It would be quite natural to assume that some spots can be more favorable and others less favorable. Examples of factors that contribute to fitness are sunlight, proximity of water, soil quality, the presence of rocks etc. Now, let us suppose that a mutant reacts differently to the same variations in the environment. Assume that a plant species does not well tolerate the presence of rocks in the soil, and that a mutant plant is more tolerant to the presence of rocks, but is very sensitive to the sunlight. Then the fitness values of the two subspecies on a spatial grid will be different, and defined by the location and by the mutation status, as assumed in our model. In the extreme case, wild type fitness is only defined by the absence of rocks, and mutant fitness is only defined by sunshine. In this case, the two fitness value sets are uncorrelated. If both factors play a role, but to different degrees, in the plants’ fitness values, then the fitness values will exhibit a degree of correlation, as described in this study.

Similar considerations apply to a large variety of biological settings. The effects of spatial structure and heterogeneity are important in biological models such as bacterial growth, where fitness can be a function of the spatial distribution of nutrients and microenvironment. In [33] it was demonstrated clearly that in biofilms, there are significant spatial microscale heterogeneities, both in chemical and physical parameters of the biofilm interstitial fluid. For example, complex patterns of water flow with different velocities and directions were observed throughout the biofilms. Further, heterogeneity in solute chemistry that is present within a biofilm was reported including concentration gradients of metabolic substrates and products. It was also reported that microorganisms within the biofilms can and do respond to these local environmental conditions in a variety ways, such as altering gene-expression patterns or physiological activities. Mutations arising and spreading in bacterial populations lead to high levels of genotypic and phenotypic heterogeneity in biofilms. It has been proposed that such diversification of bacterial populations may be considered an adaptation to the microscopic scale heterogeneity of the environment [34, 35], as different phenotypes respond differently to the changes in the environment. Diverse populations have been described as more robust; the “insurance hypothesis” states that the presence of diverse subpopulations increases the range of conditions in which the community as a whole can survive. From the theoretical prospective it is therefore essential to understand the evolutionary dynamics of mutations in the environment characterized by microscale heterogeneities.

Another important application of our theory is dynamics of cancer cell populations. It is well known that solid cancers are characterized by a highly complex and heterogeneous microenvironment [36], which includes stroma, necrotic cells, blood vessels, etc. The distribution of oxygen and hypoxic regions is highly non-homogeneous [37], the nutrients are distributed in a complex fashion, and in general, no two tumors are the same [38]. Tumours have been compared to unhealed wounds [39], in that they produce large amounts of inflammatory mediators (cytokines, chemokines, and growth factors). These molecules attract the so called tumor infiltrating cells that include macrophages, myeloid-derived suppressor cells, mesenchymal stromal cells, and TIE2-expressing monocytes. Together, these populations of non-imalignant cells contribute to the formation of a rich and heterogeneous tumor microenvironment [40]. In order to understand selection and mutation dynamics of cancer cell populations in such an environment, it is not enough to restrict the modeling efforts to the classic problems, where all the wild type cells are exactly (phenotypically) the same and all the mutant cells have the same constant fitness value. In this study, we make a step towards a more realistic view of cancer dynamics, where fitness values of different genotypes are subject to microenvironmental variations.

In our study, we consider several different scenarios, where we vary assumptions on the probability distributions underlying the mutant and wild type fitness values. In particular, we investigate the cases where

- mutant and wild type fitness probability distributions are identical (focusing on the scenarios where they are symmetric or skewed);
- mutant and wild type fitness realizations come from the same distribution and can be correlated, uncorrelated, or anti-correlated;
- the fitness distributions are different, such that one of the types is deterministic and the other random, but the mean fitness values are the same;
- the fitness distributions are different, such that one of the types is advantageous (has a larger mean fitness).

All scenarios are investigated in the context of two types of networks: circles and complete graphs. We find that the results are very different for these two choices of the underlying network.

It turns out that environmental randomness has a significant effect on the conditional fixation time of mutants. A clear trend was observed when studying the behavior of mutants on the two different networks: randomness delayed fixation of mutants on circles (at least for values of *N* larger than a threshold), and it accelerated fixation on complete graphs. The reason is that for 1D—type structures (circles), “dead zones” that form randomly in the presence of environmental influences, can significantly delay fixation by blocking the paths to fixation. For fully connected graphs, “lucky paths” form at random, that facilitate fixation. These trends have been observed for all the scenarios above, except for very small circular networks (*N* ≤ 5) some additional complexity was present. Otherwise, this pattern was universal and included scenarios with identical and different fitness probability distributions for mutants and wild type cells, in the presence of a deterministic type, and in the presence of advantageous/disadvantageous types.

Next, when studying the effects of correlations, we observed that in the case of both circles and complete graphs, the mean conditional fixation time was the largest in the fully correlated case and the smallest in the anti-correlated case.

Finally, if the probability distribution underlying the fitness realizations is skewed, large negative skewness values increase the effect of randomness on the mean conditional fixation time. This is observed in all the scenarios, whether the effect was accelerating or decelerating. Large negative skewness implies the existence of rare but very disadvantageous spots. These spots can serve as a serious impediment in the constrained circular geometry. In complete graphs, they do not present a problem because of the existence of multiple paths to fixation; at the same time having most spots with a slightly elevated fitness facilitates fixation.

The difference between fixation probabilities in circular and complete graphs exemplifies the general phenomenon of well-mixedness and its role in evolutionary mutant dynamics; it is further related to the role of dimensionality in system dynamics. Circular graphs are one-dimensional systems, where the geometric or spatial constraints are the most rigid. The opposite scenario is presented by the complete graphs, which correspond to the mass-action or complete mixing assumption. The spatially arranged two- and three-dimensional grids are somewhere between these two extreme scenarios, with the three-dimensional arrangement being closer to mass-action. We have studied evolutionary dynamics in these different settings in several contexts. For example, it has been shown that inactivation of a tumor-suppressor gene (a two-hit evolutionary process in which the cells must first become less fit before becoming more fit) happens faster in 1D (a row of cells) [41, 42], than in 2D (a layer), and this is in turn faster than in a fully mixed system with no spatial constraints [41, 43–45]. By contrast, in two-step processes in which the intermediate mutant confers a slight selective advantage, the relationship is the opposite, and a non-spatial, fully mixed environment promotes the fastest pace of evolution [45]. As was commented in [46], these phenomena seem less surprising if one notes how reminiscent they are of other fundamental laws of nature in which space dimensionality changes how things work, such as the different fundamental solutions of Poisson’s equations in 1D and 2D.

We have presented results for both small *N* and large populations. We anticipate that there are interesting applications even for small *N*. For instance, an important biological application of the ring geometry is the model of a human colonic crypt, where the relatively small (of the order of ten cells or less) population of the stem cells is situated along circular bands [47], which can be viewed as cross-sections of three-dimensional crypts. In this context, fixation is referred to as monoclonal conversion. Although the details of the exact composition of the colonic crypt is still being debated, many researchers believe that the active stem cells occupy a narrow layer, and divide mostly symmetrically. The two division types, proliferation and differentiation, are mathematically equivalent to divisions and deaths in our models. The origins of colon cancer can be studied by examining selection dynamics of mutants in such a system.

Very interesting and non-trivial is the connection between fixation time and fixation probability. These two measures of mutant success may be positively or negatively correlated for different graph structures. In particular, it appears that for a circle, randomness makes fixation longer, but it also makes it more likely; for the complete graph, randomness makes fixation both faster and more likely [22]. Counterintuitive properties of the fixation time in network structured populations were already noticed in [20]. This paper suggested that there was no obvious relation between the fixation probability and fixation time for a mutant on a network. Based on small networks, the authors analytically showed that: (i) Although the fixation probability was the same for all regular graphs (for example, circle and diamond), it would take different times for a single mutant to get fixated on these kinds of networks. (ii) The graphs that were amplifiers of selections (for example, star or line), increased the fixation probability of the mutant, but at the same time they could slow down the fixation process.

## Supporting information

S1 Text A document containing additional calculations, numerical simulations, and figures, that further illustrate points made in the main text. (PDF)
