# Supplementary material for: The effect of spatial randomness on the average fixation time of mutants
Source: PLoS Comput Biol. 2017 Nov 27;13(11):e1005864. doi: 10.1371/journal.pcbi.1005864 (PMC5720826; doi:10.1371/journal.pcbi.1005864)
Supplement: S1 Text — (PDF) [file pcbi.1005864.s001.pdf]

Text S1

The effect of spatial randomness on the average fixation time of mutants

S. Farhang-Sardroodie, A. H. Darooneh, M. Nikbakht, N. L. Komarova, M. Kohandel

Contents

1 The case $N = 4$ 1
1.1 Mean conditional fixation time for a circle with $N = 4$ 1
1.2 Mean conditional fixation time for the complete graph with $N = 4$ 4

2 The matrix method 7

3 Extension to other constant population processes 7
3.1 The birth-death formulation of Moran model 7
3.2 The Wright-Fisher model 7

4 The effect of skewness of the fitness distributions 8

5 Further results for the timing of mutant dynamics 10
5.1 Unconditional absorption time 10
5.2 The distribution of fixation time 11

1 The case $N = 4$

1.1 Mean conditional fixation time for a circle with $N = 4$

We study the evolution of a single mutant on a 4 node-circle with 14 transient states, in the context of the DB Moran process. We have four different fixation probabilities ($\rho_{n_1n_2n_3n_4}$) and times ($\tau_{n_1n_2n_3n_4}$) for one, two, three and four mutants. The recursive equations for

fixation probabilities are as follows:

$$
\begin{aligned}
4\rho_{1000} &= \frac{\tilde{a}}{\tilde{a}+c}\rho_{1100} + \frac{\tilde{a}}{\tilde{a}+c}\rho_{1001} + (\frac{\tilde{a}+3c}{\tilde{a}+c})\rho_{1000}, \\
4\rho_{0100} &= \frac{\tilde{b}}{\tilde{b}+d}\rho_{1100} + \frac{\tilde{b}}{\tilde{b}+d}\rho_{0110} + (\frac{\tilde{b}+3d}{\tilde{b}+d})\rho_{0100}, \\
4\rho_{0010} &= \frac{\tilde{c}}{\tilde{c}+a}\rho_{0110} + \frac{\tilde{c}}{\tilde{c}+a}\rho_{0011} + (\frac{\tilde{c}+3a}{\tilde{c}+a})\rho_{0010}, \\
4\rho_{0001} &= \frac{\tilde{d}}{\tilde{d}+b}\rho_{1001} + \frac{\tilde{d}}{\tilde{d}+b}\rho_{0011} + (\frac{\tilde{d}+3b}{\tilde{d}+b})\rho_{0001}, \\
2\rho_{1100} &= \frac{c}{\tilde{a}+c}\rho_{1000} + \frac{d}{\tilde{b}+d}\rho_{0100} + \frac{\tilde{b}}{\tilde{b}+d}\rho_{1110} + \frac{\tilde{a}}{\tilde{a}+c}\rho_{1101}, \\
2\rho_{1001} &= \frac{c}{\tilde{a}+c}\rho_{1000} + \frac{b}{\tilde{d}+b}\rho_{0001} + \frac{\tilde{a}}{\tilde{a}+c}\rho_{1101} + \frac{\tilde{d}}{\tilde{d}+b}\rho_{1011}, \\
2\rho_{0011} &= \frac{a}{\tilde{c}+a}\rho_{0010} + \frac{b}{\tilde{d}+b}\rho_{0001} + \frac{\tilde{d}}{\tilde{d}+b}\rho_{1011} + \frac{\tilde{c}}{\tilde{c}+a}\rho_{0111}, \\
2\rho_{0110} &= \frac{d}{\tilde{b}+d}\rho_{0100} + \frac{a}{\tilde{c}+a}\rho_{0010} + \frac{\tilde{b}}{\tilde{b}+d}\rho_{1110} + \frac{\tilde{c}}{\tilde{c}+a}\rho_{0111}, \\
4\rho_{1110} &= \frac{d}{\tilde{b}+d}\rho_{1100} + \frac{d}{\tilde{b}+d}\rho_{0110} + (\frac{d+3\tilde{b}}{d+\tilde{b}})\rho_{1110} + 1, \\
4\rho_{1101} &= \frac{c}{\tilde{a}+c}\rho_{1100} + \frac{c}{\tilde{a}+c}\rho_{1001} + (\frac{c+3\tilde{a}}{c+\tilde{a}})\rho_{1101} + 1, \\
4\rho_{1011} &= \frac{b}{\tilde{d}+b}\rho_{1001} + \frac{b}{\tilde{d}+b}\rho_{0011} + (\frac{b+3\tilde{d}}{b+\tilde{d}})\rho_{1011} + 1, \\
4\rho_{0111} &= \frac{a}{\tilde{c}+a}\rho_{0110} + \frac{a}{\tilde{c}+a}\rho_{0011} + (\frac{a+3\tilde{c}}{a+\tilde{c}})\rho_{0111} + 1. \qquad (1)
\end{aligned}
$$

Although the recursive equations for the fixation probabilities are independent, fixation probabilities also appear in the equations for the fixation times:

$$
\begin{aligned}
4\rho_{1000} &= -\frac{\tilde{a}}{\tilde{a}+c}\tau_{1100} - \frac{\tilde{a}}{\tilde{a}+c}\tau_{1001} + (\frac{3\tilde{a}+c}{\tilde{a}+c})\tau_{1000}, \\
4\rho_{0100} &= -\frac{\tilde{b}}{\tilde{b}+d}\tau_{1100} - \frac{\tilde{b}}{\tilde{b}+d}\tau_{0110} + (\frac{3\tilde{b}+d}{\tilde{b}+d})\tau_{0100}, \\
4\rho_{0010} &= -\frac{\tilde{c}}{\tilde{c}+a}\tau_{0110} - \frac{\tilde{c}}{\tilde{c}+a}\tau_{0011} + (\frac{3\tilde{c}+a}{\tilde{c}+a})\tau_{0010}, \\
4\rho_{0001} &= -\frac{\tilde{d}}{\tilde{d}+b}\tau_{1001} - \frac{\tilde{d}}{\tilde{d}+b}\tau_{0011} + (\frac{3\tilde{d}+b}{\tilde{d}+b})\tau_{0001}, \\
4\rho_{1100} &= -\frac{c}{\tilde{a}+c}\tau_{1000} - \frac{d}{\tilde{b}+d}\tau_{0100} - \frac{\tilde{b}}{\tilde{b}+d}\tau_{1110} - \frac{\tilde{a}}{\tilde{a}+c}\tau_{1101} + 2\tau_{1100}, \\
4\rho_{1001} &= -\frac{c}{\tilde{a}+c}\tau_{1000} - \frac{b}{\tilde{d}+b}\tau_{0001} - \frac{\tilde{a}}{\tilde{a}+c}\tau_{1101} - \frac{\tilde{d}}{\tilde{d}+b}\tau_{1011} + 2\tau_{1001}, \\
4\rho_{0011} &= -\frac{a}{\tilde{c}+a}\tau_{0010} - \frac{b}{\tilde{d}+b}\tau_{0001} - \frac{\tilde{d}}{\tilde{d}+b}\tau_{1011} - \frac{\tilde{c}}{\tilde{c}+a}\tau_{0111} + 2\tau_{0011}, \\
4\rho_{0110} &= -\frac{d}{\tilde{b}+d}\tau_{0100} - \frac{a}{\tilde{c}+a}\tau_{0010} - \frac{\tilde{b}}{\tilde{b}+d}\tau_{1110} - \frac{\tilde{c}}{\tilde{c}+a}\tau_{0111} + 2\tau_{0110}, \\
4\rho_{1110} &= -\frac{d}{\tilde{b}+d}\rho_{1100} - \frac{d}{\tilde{b}+d}\tau_{0110} + (\frac{3d+\tilde{b}}{d+\tilde{b}})\tau_{1110}, \\
4\rho_{1101} &= -\frac{c}{\tilde{a}+c}\tau_{1100} - \frac{c}{\tilde{a}+c}\tau_{1001} + (\frac{3c+\tilde{a}}{c+\tilde{a}})\tau_{1101}, \\
4\rho_{1011} &= -\frac{b}{\tilde{d}+b}\tau_{1001} - \frac{b}{\tilde{d}+b}\tau_{0011} + (\frac{3b+\tilde{d}}{b+\tilde{d}})\tau_{1011}, \\
4\rho_{0111} &= -\frac{a}{\tilde{c}+a}\tau_{0110} - \frac{a}{\tilde{c}+a}\tau_{0011} + (\frac{3a+\tilde{c}}{a+\tilde{c}})\tau_{0111}.
\end{aligned}
\tag{2}
$$

The mean time needed for a mutant to overtake the whole population is the average of the fixation times starting with one mutant, e.g. τ_{1000}/ρ_{1000}, taken over all fitness landscape realizations.

1.2 Mean conditional fixation time for the complete graph with $N = 4$

The Kolmogorov backward equations for the fixation probabilities on a complete graph with 16 transient states are given by,

$$
\begin{aligned}
4\rho_{1000} &= \frac{\tilde{a}}{\tilde{a}+c+d}\rho_{1100} + \frac{\tilde{a}}{\tilde{a}+c+b}\rho_{1001} + \frac{\tilde{a}}{\tilde{a}+d+b}\rho_{1010} \\
&+(\frac{c+d}{\tilde{a}+c+d} + \frac{c+b}{\tilde{a}+c+b} + \frac{b+d}{\tilde{a}+d+b})\rho_{1000}, \\
4\rho_{0100} &= \frac{\tilde{b}}{\tilde{b}+d+c}\rho_{1100} + \frac{\tilde{b}}{\tilde{b}+d+a}\rho_{0110} + \frac{\tilde{b}}{\tilde{b}+c+a}\rho_{0101} \\
&+(\frac{c+a}{\tilde{b}+c+a} + \frac{c+d}{\tilde{b}+c+d} + \frac{a+d}{\tilde{b}+d+a})\rho_{0100}, \\
4\rho_{0010} &= \frac{\tilde{c}}{\tilde{c}+a+d}\rho_{0110} + \frac{\tilde{c}}{\tilde{c}+a+b}\rho_{0011} + \frac{\tilde{c}}{\tilde{c}+d+b}\rho_{1010} \\
&+(\frac{a+d}{\tilde{c}+a+d} + \frac{a+b}{\tilde{c}+a+b} + \frac{b+d}{\tilde{c}+d+b})\rho_{0010}, \\
4\rho_{0001} &= \frac{\tilde{d}}{\tilde{d}+b+c}\rho_{1001} + \frac{\tilde{d}}{\tilde{d}+b+a}\rho_{0011} + \frac{\tilde{d}}{\tilde{d}+c+a}\rho_{0101} \\
&+(\frac{a+c}{\tilde{d}+a+c} + \frac{b+c}{\tilde{d}+b+c} + \frac{a+b}{\tilde{d}+b+a})\rho_{0001}, \\
4\rho_{1100} &= \frac{c+d}{\tilde{a}+c+d}\rho_{1000} + \frac{c+d}{\tilde{b}+d+c}\rho_{0100} + \frac{\tilde{a}+\tilde{b}}{\tilde{b}+\tilde{a}+d}\rho_{1110} + \frac{\tilde{a}+\tilde{b}}{\tilde{a}+\tilde{b}+c}\rho_{1101} \\
&+(\frac{\tilde{a}}{\tilde{a}+c+d} + \frac{\tilde{b}}{\tilde{b}+d+c} + \frac{d}{\tilde{b}+\tilde{a}+d} + \frac{c}{\tilde{a}+\tilde{b}+c})\rho_{1100}, \\
4\rho_{1001} &= \frac{c+b}{\tilde{d}+c+b}\rho_{0001} + \frac{c+b}{\tilde{a}+b+c}\rho_{1000} + \frac{\tilde{a}+\tilde{d}}{\tilde{a}+\tilde{d}+c}\rho_{1101} + \frac{\tilde{a}+\tilde{d}}{\tilde{a}+\tilde{d}+b}\rho_{1011} \\
&+(\frac{\tilde{a}}{\tilde{a}+c+b} + \frac{\tilde{d}}{\tilde{d}+b+c} + \frac{b}{\tilde{d}+\tilde{a}+b} + \frac{c}{\tilde{a}+\tilde{d}+c})\rho_{1001}, \\
4\rho_{0110} &= \frac{a+d}{\tilde{b}+a+d}\rho_{0100} + \frac{a+d}{\tilde{c}+d+a}\rho_{0010} + \frac{\tilde{b}+\tilde{c}}{\tilde{b}+\tilde{c}+d}\rho_{1110} + \frac{\tilde{b}+\tilde{c}}{\tilde{b}+\tilde{c}+a}\rho_{0111} \\
&+(\frac{\tilde{b}}{\tilde{b}+a+d} + \frac{\tilde{c}}{\tilde{c}+d+a} + \frac{d}{\tilde{b}+\tilde{c}+d} + \frac{a}{\tilde{c}+\tilde{b}+a})\rho_{0110}, \\
4\rho_{0011} &= \frac{a+b}{\tilde{c}+a+b}\rho_{0010} + \frac{a+b}{\tilde{d}+a+b}\rho_{0001} + \frac{\tilde{c}+\tilde{d}}{\tilde{c}+\tilde{d}+b}\rho_{1011} + \frac{\tilde{c}+\tilde{d}}{\tilde{c}+\tilde{d}+a}\rho_{0111} \\
&+(\frac{\tilde{c}}{\tilde{c}+a+b} + \frac{\tilde{d}}{\tilde{d}+a+b} + \frac{b}{\tilde{c}+\tilde{d}+b} + \frac{a}{\tilde{c}+\tilde{d}+a})\rho_{0011}, \\
4\rho_{1010} &= \frac{d+b}{\tilde{a}+b+d}\rho_{1000} + \frac{d+b}{\tilde{c}+b+d}\rho_{0010} + \frac{\tilde{a}+\tilde{c}}{\tilde{a}+\tilde{c}+d}\rho_{1110} + \frac{\tilde{a}+\tilde{c}}{\tilde{a}+\tilde{c}+b}\rho_{1011} \\
&+(\frac{\tilde{a}}{\tilde{a}+b+d} + \frac{\tilde{c}}{\tilde{c}+b+d} + \frac{d}{\tilde{a}+\tilde{c}+d} + \frac{b}{\tilde{a}+\tilde{c}+b})\rho_{1010},
\end{aligned}
$$

$$
\begin{aligned}
4\rho_{0101} &= \frac{a+c}{\tilde{b}+a+c}\rho_{0100}+\frac{a+c}{\tilde{d}+a+c}\rho_{0001}+\frac{\tilde{b}+\tilde{d}}{\tilde{b}+\tilde{d}+a}\rho_{0111}+\frac{\tilde{b}+\tilde{d}}{\tilde{b}+\tilde{d}+c}\rho_{1101} \\
&\quad +(\frac{\tilde{b}}{\tilde{b}+a+c}+\frac{\tilde{d}}{\tilde{d}+a+c}+\frac{a}{\tilde{b}+\tilde{d}+a}+\frac{c}{\tilde{b}+\tilde{d}+c})\rho_{1010}, \\
4\rho_{1110} &= \frac{d}{\tilde{b}+\tilde{a}+d}\rho_{1100}+\frac{d}{\tilde{b}+\tilde{c}+d}\rho_{0110}+\frac{d}{\tilde{a}+\tilde{c}+d}\rho_{1010} \\
&\quad +(\frac{\tilde{a}+\tilde{c}}{\tilde{a}+\tilde{c}+d}+\frac{\tilde{a}+\tilde{b}}{\tilde{a}+\tilde{b}+d}+\frac{\tilde{b}+\tilde{c}}{\tilde{b}+\tilde{c}+d})\rho_{1110}+1, \\
4\rho_{1101} &= \frac{c}{\tilde{b}+\tilde{a}+c}\rho_{1100}+\frac{c}{\tilde{a}+\tilde{d}+c}\rho_{1001}+\frac{c}{\tilde{b}+\tilde{d}+c}\rho_{0101} \\
&\quad +(\frac{\tilde{a}+\tilde{b}}{\tilde{a}+\tilde{b}+c}+\frac{\tilde{a}+\tilde{d}}{\tilde{a}+\tilde{d}+c}+\frac{\tilde{b}+\tilde{d}}{\tilde{b}+\tilde{d}+c})\rho_{1101}+1, \\
4\rho_{1011} &= \frac{b}{\tilde{d}+\tilde{a}+b}\rho_{1001}+\frac{b}{\tilde{d}+\tilde{c}+b}\rho_{0011}+\frac{b}{\tilde{a}+\tilde{c}+b}\rho_{1010} \\
&\quad +(\frac{\tilde{a}+\tilde{d}}{\tilde{a}+\tilde{d}+b}+\frac{\tilde{c}+\tilde{d}}{\tilde{c}+\tilde{d}+b}+\frac{\tilde{c}+\tilde{a}}{\tilde{a}+\tilde{c}+b})\rho_{1011}+1, \\
4\rho_{0111} &= \frac{a}{\tilde{b}+\tilde{c}+a}\rho_{0110}+\frac{a}{\tilde{d}+\tilde{c}+a}\rho_{0011}+\frac{a}{\tilde{b}+\tilde{d}+a}\rho_{0101} \\
&\quad +(\frac{\tilde{b}+\tilde{c}}{\tilde{b}+\tilde{c}+a}+\frac{\tilde{d}+\tilde{c}}{\tilde{d}+\tilde{c}+a}+\frac{\tilde{b}+\tilde{d}}{\tilde{b}+\tilde{d}+a})\rho_{0111}+1.
\end{aligned} \tag{3}
$$

In addition, the equations for the fixation time are given by

$$
\begin{aligned}
4\rho_{1000} &= -\frac{\tilde{a}}{\tilde{a}+c+d}\tau_{1100}-\frac{\tilde{a}}{\tilde{a}+c+b}\tau_{1001}-\frac{\tilde{a}}{\tilde{a}+d+b}\tau_{1010} \\
&\quad +(1+\frac{\tilde{a}}{\tilde{a}+c+d}+\frac{\tilde{a}}{\tilde{a}+c+b}+\frac{\tilde{a}}{\tilde{a}+d+b})\tau_{1000}, \\
4\rho_{0100} &= -\frac{\tilde{b}}{\tilde{b}+d+c}\tau_{1100}-\frac{\tilde{b}}{\tilde{b}+d+a}\tau_{0110}-\frac{\tilde{b}}{\tilde{b}+c+a}\tau_{0101} \\
&\quad +(1+\frac{\tilde{b}}{\tilde{b}+c+a}+\frac{\tilde{b}}{\tilde{b}+c+d}+\frac{\tilde{b}}{\tilde{b}+d+a})\tau_{0100}, \\
4\rho_{0010} &= -\frac{\tilde{c}}{\tilde{c}+a+d}\tau_{0110}-\frac{\tilde{c}}{\tilde{c}+a+b}\tau_{0011}-\frac{\tilde{c}}{\tilde{c}+d+b}\tau_{1010} \\
&\quad +(1+\frac{\tilde{c}}{\tilde{c}+a+d}+\frac{\tilde{c}}{\tilde{c}+a+b}+\frac{\tilde{c}}{\tilde{c}+d+b})\tau_{0010}, \\
4\rho_{0001} &= -\frac{\tilde{d}}{\tilde{d}+b+c}\tau_{1001}-\frac{\tilde{d}}{\tilde{d}+b+a}\tau_{0011}-\frac{\tilde{d}}{\tilde{d}+c+a}\tau_{0101} \\
&\quad +(1+\frac{\tilde{d}}{\tilde{d}+a+c}+\frac{\tilde{d}}{\tilde{d}+b+c}+\frac{\tilde{d}}{\tilde{d}+b+a})\tau_{0001},
\end{aligned}
$$

$$\begin{aligned}
4\rho_{1100} &= -\frac{c+d}{\tilde{a}+c+d}\tau_{1000}-\frac{c+d}{\tilde{b}+d+c}\tau_{0100}-\frac{\tilde{a}+\tilde{b}}{\tilde{b}+\tilde{a}+d}\tau_{1110}-\frac{\tilde{a}+\tilde{b}}{\tilde{a}+\tilde{b}+c}\tau_{1101} \\
&\quad +(\frac{c+d}{\tilde{a}+c+d}+\frac{c+d}{\tilde{b}+d+c}+\frac{\tilde{a}+\tilde{b}}{\tilde{b}+\tilde{a}+d}+\frac{\tilde{a}+\tilde{b}}{\tilde{a}+\tilde{b}+c})\tau_{1100}, \\
4\rho_{1001} &= -\frac{c+b}{\tilde{d}+c+b}\tau_{0001}-\frac{c+b}{\tilde{a}+b+c}\tau_{1000}-\frac{\tilde{a}+\tilde{d}}{\tilde{a}+\tilde{d}+c}\tau_{1101}-\frac{\tilde{a}+\tilde{d}}{\tilde{a}+\tilde{d}+b}\tau_{1011} \\
&\quad +(\frac{b+c}{\tilde{a}+c+b}+\frac{b+c}{\tilde{d}+b+c}+\frac{\tilde{a}+\tilde{d}}{\tilde{d}+\tilde{a}+b}+\frac{\tilde{a}+\tilde{d}}{\tilde{a}+\tilde{d}+c})\tau_{1001}, \\
4\rho_{0110} &= -\frac{a+d}{\tilde{b}+a+d}\tau_{0100}-\frac{a+d}{\tilde{c}+d+a}\tau_{0010}-\frac{\tilde{b}+\tilde{c}}{\tilde{b}+\tilde{c}+d}\tau_{1110}-\frac{\tilde{b}+\tilde{c}}{\tilde{b}+\tilde{c}+a}\tau_{0111} \\
&\quad +(\frac{a+d}{\tilde{b}+a+d}+\frac{a+d}{\tilde{c}+d+a}+\frac{\tilde{b}+\tilde{c}}{\tilde{b}+\tilde{c}+d}+\frac{\tilde{b}+\tilde{c}}{\tilde{c}+\tilde{b}+a})\tau_{0110}, \\
4\rho_{0011} &= -\frac{a+b}{\tilde{c}+a+b}\tau_{0010}-\frac{a+b}{\tilde{d}+a+b}\tau_{0001}-\frac{\tilde{c}+\tilde{d}}{\tilde{c}+\tilde{d}+b}\tau_{1011}-\frac{\tilde{c}+\tilde{d}}{\tilde{c}+\tilde{d}+a}\tau_{0111} \\
&\quad +(\frac{a+b}{\tilde{c}+a+b}+\frac{a+b}{\tilde{d}+a+b}+\frac{\tilde{c}+\tilde{d}}{\tilde{c}+\tilde{d}+b}+\frac{\tilde{c}+\tilde{d}}{\tilde{c}+\tilde{d}+a})\tau_{0011}, \\
4\rho_{1010} &= -\frac{d+b}{\tilde{a}+b+d}\tau_{1000}-\frac{d+b}{\tilde{c}+b+d}\tau_{0010}-\frac{\tilde{a}+\tilde{c}}{\tilde{a}+\tilde{c}+d}\tau_{1110}-\frac{\tilde{a}+\tilde{c}}{\tilde{a}+\tilde{c}+b}\tau_{1011} \\
&\quad +(\frac{b+d}{\tilde{a}+b+d}+\frac{b+d}{\tilde{c}+b+d}+\frac{\tilde{a}+\tilde{c}}{\tilde{a}+\tilde{c}+d}+\frac{\tilde{a}+\tilde{c}}{\tilde{a}+\tilde{c}+b})\tau_{1010}, \\
4\rho_{0101} &= -\frac{a+c}{\tilde{b}+a+c}\tau_{0100}-\frac{a+c}{\tilde{d}+a+c}\tau_{0001}-\frac{\tilde{b}+\tilde{d}}{\tilde{b}+\tilde{d}+a}\tau_{0111}-\frac{\tilde{b}+\tilde{d}}{\tilde{b}+\tilde{d}+c}\tau_{1101} \\
&\quad +(\frac{a+c}{\tilde{b}+a+c}+\frac{a+c}{\tilde{d}+a+c}+\frac{\tilde{b}+\tilde{d}}{\tilde{b}+\tilde{d}+a}+\frac{\tilde{b}+\tilde{d}}{\tilde{b}+\tilde{d}+c})\tau_{1010}, \\
4\rho_{1110} &= -\frac{d}{\tilde{b}+\tilde{a}+d}\tau_{1100}-\frac{d}{\tilde{b}+\tilde{c}+d}\tau_{0110}-\frac{d}{\tilde{a}+\tilde{c}+d}\tau_{1010} \\
&\quad +(1+\frac{d}{\tilde{a}+\tilde{c}+d}+\frac{d}{\tilde{a}+\tilde{b}+d}+\frac{d}{\tilde{b}+\tilde{c}+d})\tau_{1110}, \\
4\rho_{1101} &= -\frac{c}{\tilde{b}+\tilde{a}+c}\tau_{1100}-\frac{c}{\tilde{a}+\tilde{d}+c}\tau_{1001}-\frac{c}{\tilde{b}+\tilde{d}+c}\tau_{0101} \\
&\quad +(1+\frac{c}{\tilde{a}+\tilde{b}+c}+\frac{c}{\tilde{a}+\tilde{d}+c}+\frac{c}{\tilde{b}+\tilde{d}+c})\tau_{1101}, \\
4\rho_{1011} &= -\frac{b}{\tilde{d}+\tilde{a}+b}\tau_{1001}-\frac{b}{\tilde{d}+\tilde{c}+b}\tau_{0011}-\frac{b}{\tilde{a}+\tilde{c}+b}\tau_{1010} \\
&\quad +(1+\frac{b}{\tilde{a}+\tilde{d}+b}+\frac{b}{\tilde{c}+\tilde{d}+b}+\frac{b}{\tilde{a}+\tilde{c}+b})\tau_{1011}, \\
4\rho_{0111} &= -\frac{a}{\tilde{b}+\tilde{c}+a}\tau_{0110}-\frac{a}{\tilde{d}+\tilde{c}+a}\tau_{0011}-\frac{a}{\tilde{b}+\tilde{d}+a}\tau_{0101} \\
&\quad +(1+\frac{a}{\tilde{b}+\tilde{c}+a}+\frac{a}{\tilde{d}+\tilde{c}+a}+\frac{a}{\tilde{b}+\tilde{d}+a})\tau_{0111}.
\end{aligned} \tag{4}$$

2 The matrix method

Following the method described in [1, 2, 3], one can also use the canonical form of the transition matrix, to find the same exact solutions for the evolutionary properties of a mutant, as were found in the main text. For a Markov chain with r absorbing states and t transient states, such that $r + t = s$ is the total number of states, the transition matrix has the following canonical form

$$T_{s\times s} = \left(\begin{array}{c|c} Q & R \\ \hline O & I \end{array}\right),$$

where Q is a $t \times t$ matrix of transition probabilities between transient states and R is a $t \times r$ matrix which describes transitions from the transient states into absorbing states. I is the $r \times r$ identity matrix, which indicates that once the process gets absorbed, it will be there forever. The matrix O is a $r \times t$ zero matrix that shows that there is no transition from absorbing states to transient states. For the three-node graph with variable fitness at each vertex, we can generally write the following canonical matrix between transition states

$$T = \left(\begin{array}{c|cccccc|cc}
\text{states} & (100) & (010) & (001) & (101) & (110) & (011) & (000) & (111) \\ \hline
100 & \frac{b}{3(\tilde{a}+b)} + \frac{c}{3(\tilde{a}+c)} & 0 & 0 & \frac{\tilde{a}}{3(\tilde{a}+b)} & \frac{\tilde{a}}{3(\tilde{a}+c)} & 0 & \frac{1}{3} & 0 \\
010 & 0 & \frac{c}{3(\tilde{b}+c)} + \frac{a}{3(\tilde{b}+a)} & 0 & 0 & \frac{\tilde{b}}{3(\tilde{b}+c)} & \frac{\tilde{b}}{3(\tilde{b}+a)} & \frac{1}{3} & 0 \\
001 & 0 & 0 & \frac{a}{3(\tilde{c}+a)} + \frac{b}{3(\tilde{c}+b)} & \frac{\tilde{c}}{3(\tilde{c}+b)} & 0 & \frac{\tilde{c}}{3(\tilde{c}+a)} & \frac{1}{3} & 0 \\
101 & \frac{b}{3(\tilde{a}+b)} & 0 & \frac{b}{3(\tilde{c}+b)} & \frac{\tilde{a}}{3(\tilde{a}+b)} + \frac{\tilde{c}}{3(\tilde{c}+b)} & 0 & 0 & 0 & \frac{1}{3} \\
110 & \frac{c}{3(\tilde{a}+c)} & \frac{c}{3(\tilde{b}+c)} & 0 & 0 & \frac{\tilde{a}}{3(\tilde{a}+c)} + \frac{\tilde{b}}{3(\tilde{b}+c)} & 0 & 0 & \frac{1}{3} \\
011 & 0 & \frac{a}{3(\tilde{b}+a)} & \frac{a}{3(\tilde{c}+a)} & 0 & 0 & \frac{\tilde{b}}{3(\tilde{b}+a)} + \frac{\tilde{c}}{3(\tilde{c}+a)} & 0 & \frac{1}{3} \\ \hline
000 & 0 & 0 & 0 & 0 & 0 & 0 & 1 & 0 \\
111 & 0 & 0 & 0 & 0 & 0 & 0 & 0 & 1
\end{array}\right). \tag{5}$$

Let matrix F be the inverse of the matrix $I - Q$ (I is a $t \times t$ identity matrix), whose entry F_{ij} is the expected time that is spent in state j, given the starting state was i. Using the formulas $\phi_{ij} = (FR)_{ij}$ and $t_i^N = \sum_{j=1}^{N-1}(\frac{\phi_{jN}}{\phi_{iN}} F_{ij})$ we can find the fixation probability for mutants [1], resulting in the same answers as those obtained using the Kolmogorov equations.

3 Extension to other constant population processes

3.1 The birth-death formulation of Moran model

In this formulation (for the complete graph), first a cell is selected for division (based on the cell's fitness), and then another cell is randomly chosen for death (with a uniform distribution), after which the progeny of the cell that divided replaces the dead cell. We assume that a cell that just divided cannot die in the same update. We have compared the results for the the death-birth (DB) and birth-death (BD) formulations of the Moran model in figure (1).

3.2 The Wright-Fisher model

We consider a haploid Wright-Fisher model, where the each generation is represented as N spots that are characterized with random fitness values (as before, fitness values remain

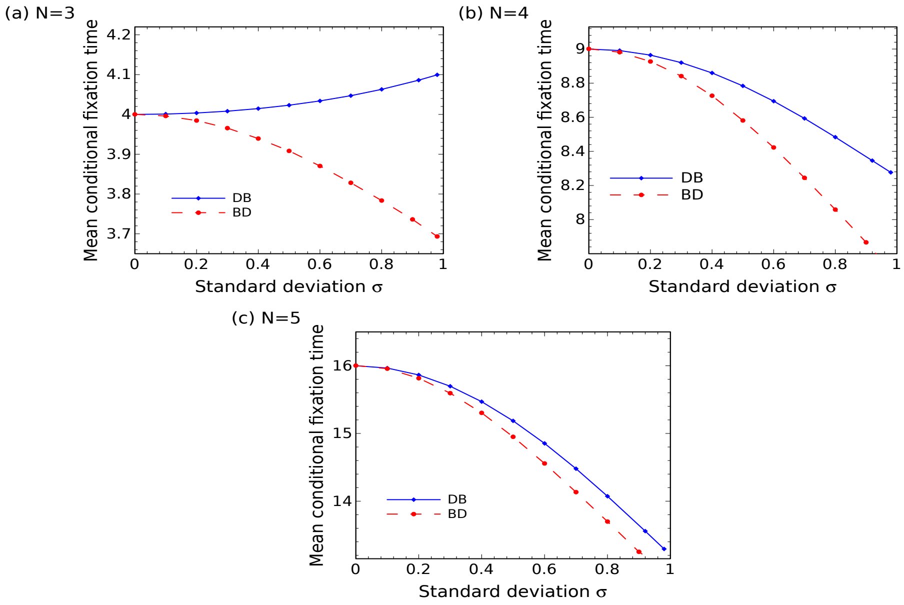

Figure 1: The mean conditional mutant fixation time (starting with one mutant) as a function of standard deviation for the DB and BD Moran models for $N = 3, 4, 5$. The points are based on stochastic simulations and the solid curves are based on the exact analytical calculations.

constant throughout each realization). To form the next generation, we randomly select (with replacement) cells from the current generation, and use their copies to populate the next generation. The mean conditional mutant fixation time in the haploid Wright-Fisher model as a function of the standard deviation is presented in figure (2).

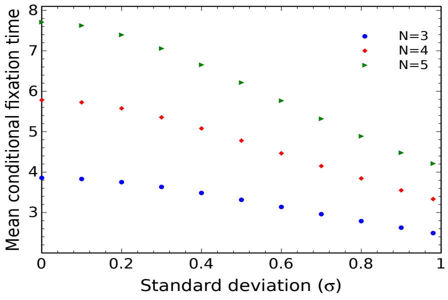

Figure 2: The mean conditional mutant fixation time (based on numerical simulations) as a function of standard deviation for the Write-Fisher model for $N = 3, 4, 5$.

4 The effect of skewness of the fitness distributions

In figure 3 of the main text, we study the effect of skewness on the timing of mutant fixation. Here we provide a more in depth analysis of the trends observed, and provide intuitive explanations.

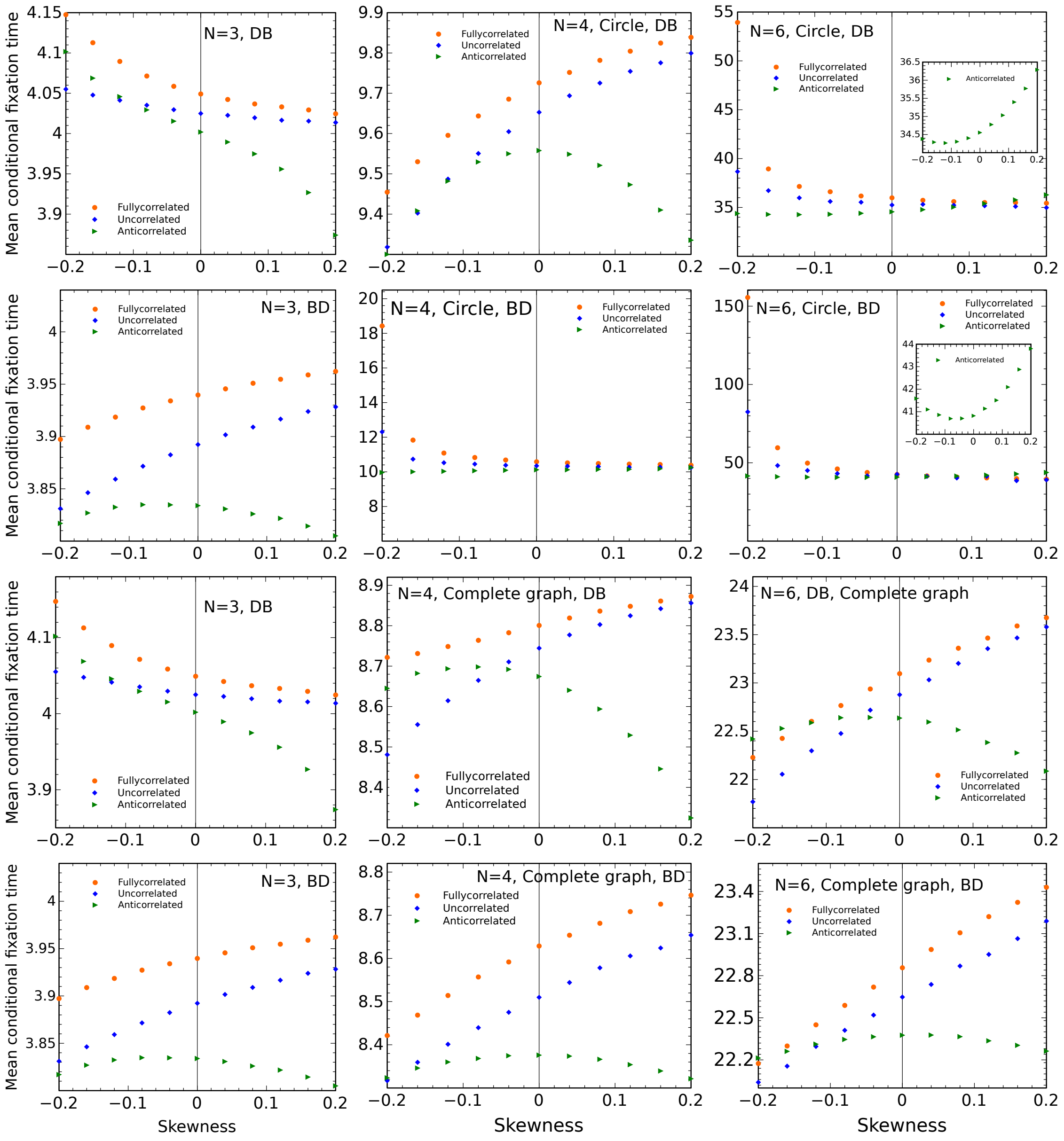

Figure 3: The mean conditional mutant fixation time as a function of skewness for the Moran model for $N = 3, 4, 6$. The rows (from top to bottom) correspond to DB on a circle, BD on a circle, DB on a complete graph, and BD on a complete graph. Three cases are shown in each graph: (orange) mutant and wild type fitness values are fully correlated; (blue) they are uncorrelated, and (green) they are anti-correlated. The two rightmost images in the 1st and 2nd rows contain insets, that show the non-monotonic behavior of the mean conditional mutant fixation time for the anti correlated cases.

One can see in figure 3 of the main text that both fully correlated and uncorrelated systems exhibit a monotonic dependence on the skewness. We have verified that the dependence remains monotonic if one expands the skewness range to larger positive values (not shown). In order to investigate the dependence on skewness further, we have examined the behavior for larger values of N, in a systematic way (that is, for 4 models: circle DB, circle BD, complete graph DB, and complete graph BD), see figure 3. We observe a pattern that we have seen repeatedly in this study: the behavior for small values of N is not always representative, but as N grows, a certain tendency emerges. In the context of the dependence of mean conditional fixation time on skewness, we observe that the differences between BD and DB diminish for larger values of N, but the behavior of circular networks is quite different from that of complete graphs. For uncorrelated and correlated fitness distributions, the time to fixation decays with skewness for circles, and increases with skewness for complete graphs.

Furthermore, we observe that for anti-correlated fitness landscapes, the non-monotonic behavior persists for large N both in the case of circles and complete graphs (interestingly, the concavity is different in the two cases). In other words, large negative and positive skewness values behave similar to each other. This phenomenon may have an intuitive explanation. Consider a fitness landscape for wild type cells that is drawn from a distribution with a large positive skewness. Such a landscape will mostly consist of fitness values x_1 that are just below 1, and occasionally, of values x_2 that are significantly greater than 1. To create the anti-correlated mutant landscape matching this wild type landscape, we replace all the values x_1 with values $2 - x_1 > 1$ (just above 1), and all the values x_2 with values $2 - x_2 < 1$. The landscapes obtained by this procedure have the same mean and variance as the original ones, and their skewness is the negative of the original skewness. This shows that by construction of anti-correlated landscapes with a nonzero skewness, a wild landscape with a positive skewness is paired up with a mutant landscape with a negative skewness (of the same absolute value). In a sense, positive and negative skewness values always coexist in such systems. Note that they are not exactly symmetric, as initially, there are $N-1$ wild type cells and only 1 mutant cell, so most cells exist on the wild-type fitness landscape. Nonetheless, because of this effect of skewness reversal as more and more mutants are created, we can see that increasing the absolute value of skewness in either direction could lead to a similar trend in behavior.

5 Further results for the timing of mutant dynamics

5.1 Unconditional absorption time

We are also interested to examine mutant fixation behavior depending on the initial number of mutants. Instead of starting with one mutant individual in a pool of wild type population, we can examine a Markov chain with different initial number of mutants. We investigate the unconditional absorption time, which is the expected time to get into either of the two absorbing states (characterized by all mutants or all wild-type cells). In figure 4 we denote the initial mutant fraction as n_0. The unconditional mean absorption time is a one-hump symmetric function of n_0 with a maximum at $n_0 = 1/2$ (since this point is the furthest from either of the absorbing states). Examining the influence of randomness, we can see that the unconditional absorption time increases with randomness for a circle (figure 4(a)) and

decreases with randomness for a complete graph (figure 4(b)), which is consistent with the results in the main text.

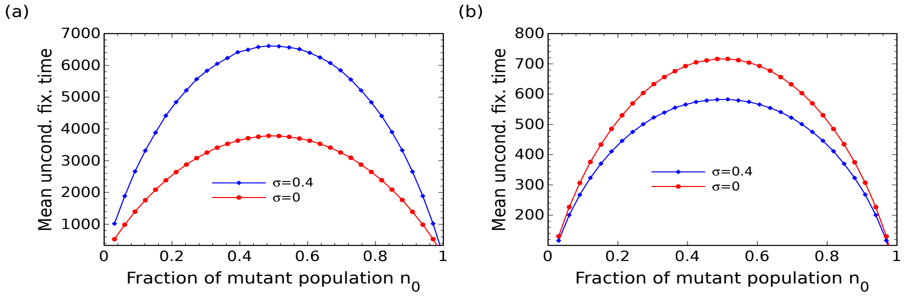

Figure 4: Unconditional absorption time for population size $N = 33$, as a function of the initial fraction of mutants. Panels (a) and (b) show the results for circles and complete graphs, respectively. Solid lines indicate analytical results (using the matrix method). Each data point is averaged over 10^6 independent realizations.

5.2 The distribution of fixation time

In figure 5, the distribution function of fixation time for a mutant is represented for $N = 4$ through $N = 6$ (panels (a) and (b)). The standard deviation of individuals' random fitness in Moran process is $\sigma = 0.7$ and its mean value is 1. We notice that the fitted distribution function corresponds to the negative binomial distribution with two parameters r and p as follow:

$$f(k; r, p) = P_N(t = k) = \binom{k + r - 1}{k} .(1 - p)^r p^k \quad , \quad k = 0, 1, 2, \cdots$$

Here $r = \frac{\mu^2}{s^2 - \mu}$ and $p = \frac{s^2 - \mu}{s^2}$, where μ and s are the mean value and the standard deviation of the conditional fixation times, respectively. In panels (a) and (b): the blue lines show the distribution function for $N = 4$, the red lines give the distribution function for $N = 6$, and the green lines corresponds to the distribution function with $N = 8$. The corresponding

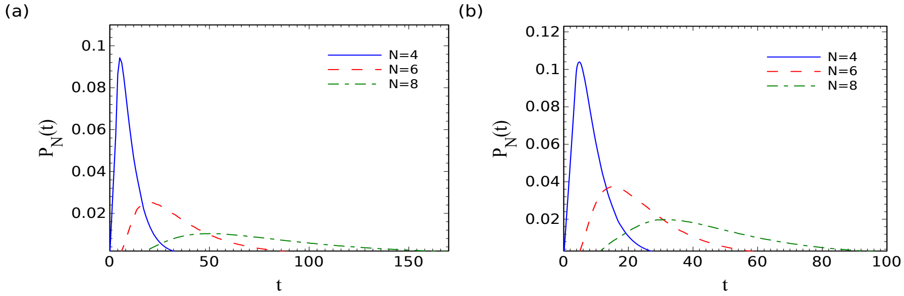

Figure 5: The distribution function of conditional fixation time for a mutant with different fitness configurations. Panel (a) and (b) show the results for circles and complete graphs with population sizes: $N = 4, 6, 8$. The standard deviation is $\sigma = 0.7$.

parameters of distribution function are given in tables 1 and 2 for circle and complete graph

with different population sizes. As expected, as N gets larger, the average time to fixation for mutant population gets larger, and also the distribution of the fixation time becomes wider.

Table 1: Circle.

Size of graph	μ	s	r	p
4	9.3425	6.1997	3	0.75694
6	27.754	20.32	2	0.93278
8	71.806	51.477	2	0.9729

Table 2: Complete graph.

Size of graph	μ	σ	r	p
4	7.6113	5.1886	3	0.71728
6	23.262	14.27	3	0.88577
8	45.04	26.856	3	0.93755

References

[1] Grinstead CM, Snell JL. Introduction to probability. American Mathematical Soc. 2012.

[2] Hindersin, L & Traulsen, A. Counterintuitive properties of the fixation time in network-structured populationson. Journal of The Royal Society Interface-The Royal Society. 2014;11(99):20140606.

[3] Hauser OP, Traulsen A, Nowak MA. Heterogeneity in background fitness acts as a suppressor of selection. Journal of theoretical biology-Elsevier. 2014;343:178–185.
